# Characterization of renal cell carcinoma‐associated constitutional chromosome abnormalities by genome sequencing

**DOI:** 10.1002/gcc.22833

**Published:** 2020-02-05

**Authors:** Philip S. Smith, James Whitworth, Hannah West, Jacqueline Cook, Carol Gardiner, Derek H. K. Lim, Patrick J. Morrison, R. Gordon Hislop, Emily Murray, NIHR Rare Disease BioResource, Marc Tischkowitz, Anne Y. Warren, Emma R. Woodward, Eamonn R. Maher

**Affiliations:** ^1^ Department of Medical Genetics University of Cambridge and NIHR Cambridge Biomedical Research Centre, Cancer Research UK Cambridge Centre, Cambridge Biomedical Campus Cambridge UK; ^2^ Department of Clinical Genetics Sheffield Children's Hospital Sheffield UK; ^3^ West of Scotland Genetics Services Queen Elizabeth University Hospital Glasgow UK; ^4^ West Midlands Regional Genetics Service Birmingham Women's and Children's National Health Service (NHS) Foundation Trust Birmingham UK; ^5^ Northern Ireland Regional Genetics Service Belfast City Hospital, Belfast Health & Social Care Trust Belfast UK; ^6^ East of Scotland Regional Genetics Service Ninewells Hospital Dundee UK; ^7^ NIHR BioResource Cambridge University Hospitals, Cambridge Biomedical Campus Cambridge UK; ^8^ Department of Histopathology Cambridge University NHS Foundation Trust and Cancer Research UK Cambridge Centre Cambridge UK; ^9^ Manchester Centre for Genomic Medicine and NW Laboratory Genetics Hub, Manchester University Hospitals NHS Foundation Trust, Division of Evolution and Genomic Sciences, School of Biological Sciences, Faculty of Biology, Medicine and Health University of Manchester, Health Innovation Manchester Manchester UK

**Keywords:** genetics, renal cell carcinoma, translocation, whole genome sequencing

## Abstract

Constitutional translocations, typically involving chromosome 3, have been recognized as a rare cause of inherited predisposition to renal cell carcinoma (RCC) for four decades. However, knowledge of the molecular basis of this association is limited. We have characterized the breakpoints by genome sequencing (GS) of constitutional chromosome abnormalities in five individuals who presented with RCC. In one individual with constitutional t(10;17)(q11.21;p11.2), the translocation breakpoint disrupted two genes: the known renal tumor suppressor gene (TSG) *FLCN* (and clinical features of Birt‐Hogg‐Dubé syndrome were detected) and *RASGEF1A*. In four cases, the rearrangement breakpoints did not disrupt known inherited RCC genes. In the second case without chromosome 3 involvement, the translocation breakpoint in an individual with a constitutional t(2;17)(q21.1;q11.2) mapped 12 Kb upstream of *NLK.* Interestingly, NLK has been reported to interact indirectly with FBXW7 and a previously reported RCC‐associated translocation breakpoint disrupted *FBXW7*. In two cases of constitutional chromosome 3 translocations, no candidate TSGs were identified in the vicinity of the breakpoints. However, in an individual with a constitutional chromosome 3 inversion, the 3p breakpoint disrupted the *FHIT* TSG (which has been reported previously to be disrupted in two apparently unrelated families with an RCC‐associated t(3;8)(p14.2;q24.1). These findings (a) expand the range of constitutional chromosome rearrangements that may be associated with predisposition to RCC, (b) confirm that chromosome rearrangements not involving chromosome 3 can predispose to RCC, (c) suggest that a variety of molecular mechanisms are involved the pathogenesis of translocation‐associated RCC, and (d) demonstrate the utility of GS for investigating such cases.

## 1 INTRODUCTION

Kidney cancer accounts for almost 2% of new cancer diagnoses globally and the incidence increased by 36% between 1990 and 2013.^1^ The most common form of kidney cancer in adults is renal cell carcinoma (RCC), which is histologically and genetically heterogeneous. Approximately 3% of cases of RCC are recognized as having a genetic basis and a variety of syndromic and non‐syndromic forms of RCC have been delineated.^2^ Although familial forms of RCC are infrequent, the identification of the molecular basis of inherited RCC, as exemplified by von Hippel‐Lindau (VHL; MIM 193300) disease, has been crucial to help understand the molecular mechanisms of sporadic cases of RCC.^3^ In addition to *VHL*, germline mutations in multiple other genes have been reported to predispose to RCC including *BAP1*, *FH*, *FLCN*, *MET*, *PTEN*, *SDHB*, *SDHD*, *SDHA*, and *SDHC*.^2^, ^4^ Furthermore constitutional translocations, particularly those involving chromosome 3, have been associated with inherited RCC in multiple reports.

Four decades ago, Cohen et al^5^ described a large kindred in which clear cell RCC segregated with a constitutional translocation between the short arm of chromosome 3 and the long arm of chromosome 8, t(3;8)(p14.2;q24.1), such that the risk of RCC in translocation carriers was estimated to be 80% at age 60 years.^5^ Subsequently somatic deletions of the short arm of chromosome 3 (3p) were found to be the most common cytogenetic abnormality in sporadic clear cell RCC suggesting the presence of critical renal tumor suppressor genes (TSGs) on 3p.^6^ These developments led to the suggestion that identification of individuals with suspected inherited forms of RCC should be screened for constitutional translocations involving 3p and that the characterization of RCC‐associated translocation breakpoints might lead to the identification of novel inherited RCC genes.^7^ Subsequent research studies have confirmed that the short arm of chromosome 3 does indeed harbor several TSGs that are frequently inactivated in sporadic RCC (eg, *VHL*, *PBRM1*, *BAP1*, and *RASSF1A*).^8^, ^9^, ^10^, ^11^, ^12^, ^13^, ^14^, ^15^

In a review of previously published reports, we identified 17 RCC‐associated constitutional translocations (15 of which involved a chromosome 3 breakpoint) of constitutional chromosome abnormalities associated with RCC (Table 1).^7^, ^16^, ^17^, ^18^, ^19^, ^20^, ^21^, ^22^, ^23^, ^24^, ^25^, ^26^, ^27^, ^28^, ^29^ Molecular characterization of the translocation breakpoints in individual cases have identified a series of candidate TSGs disrupted (or nearby) the translocation breakpoints but none of the 15 cases with chromosome 3 breakpoints was found to disrupt either 3p genes that are frequently mutated in sporadic RCC or known familial RCC genes that map outside of 3p (eg, *FLCN*, *FH*, and *SDHB*). The observation that the chromosome 3 breakpoints in RCC‐associated translocations were heterogeneous led to the suggestion that RCC predisposition in such cases might not necessarily involve disruption of a TSG but might confer susceptibility because of instability of the derivative chromosome 3 leading to loss at an early stage of tumorigenesis.^30^

**Table 1:** Clinical features of RCC in individuals from families with a constitutional chromosome rearrangement

Publication(s)	Rearrangement	Breakpoint(GRCh38)	Histology (RCC)	Type (foci = n)	Sex	Age
Cohen et al^5^	t(3;8)(pl4.2;q24.1)	N/a	Clear cell	Bilateral (n = 2)	M	37
			Clear cell	Bilateral (n = 3)	M	45
			Clear cell	Unilateral (n > 2)	M	59
			Clear cell	Unilateral (n = 3)	F	46
			Clear cell	Unilateral (n = 1)	M	44
			Clear cell	Unilateral (n = 1)	F	50
			Clear cell	Bilateral (n > 3)	F	41
			Clear cell^a^	Bilateral (n > 2)	M	47
			Clear cell^a^	Bilateral (n = 9)	F	44
			Not specified	Bilateral (n = 7)	F	39
Kovacs et al 16	t(3;12)(q13.2;q24.1)	N/a	Clear cell	Unilateral (n = 1)	M	50
Kovacs et al 16	t(3;6)(p13;q25.1)	N/a	Clear cell	Bilateral (n = 5)	M	53
Koolen et al^17^	t(2;3)(q35;q21)	N/a	Clear cell	Bilateral (n = 3)	M	54
			Not specified	N/a	F	53
			Clear cell	Unilateral (n = 3)	F	68
			Clear cell	Unilateral (n = 1)	M	40
			Clear cell	Bilateral (n = 2)	M	30
Van Kessel et al^18^	t(3;4)(p13;p16)	N/a	Clear cell	N/a	M	52
Eleveld et al^19^	t(3;6)(q11.2;q13)	N/a	Clear cell	Unilateral	F	59
			Clear cell	Unilateral	F	41
			Clear cell	Unilateral	F	63
			Clear cell	Unilateral	M	67
Kanayama et al 20	t(1;3)(q32;q13.3)	N/a	Clear cell	Unilateral (n = 1)	F	79
			Clear cell	Bilateral (n = 4)	M	56
			Clear cell^a^	Unilateral (n = 1)	M	70
			Clear cell	Unilateral (n = 1)	M	62
Podolski et al 21	t(2;3)(q33;q21)	N/a	Clear cell	N/a	M	45
			Clear cell	N/a	M	38
			Clear cell^a^	N/a	M	51
			Clear cell^a^	N/a	F	51
			Clear cell^a^	N/a	F	51
			Clear cell^a^	Bilateral	M	51
			Clear cell^a^	N/a	F	63
Meléndez et al^22^	t(3;8)(p14.1;q24.23)	N/a	Clear cell	Bilateral (n = 2)	M	46
			Clear cell	Bilateral (n = N/a)	F	56
			Clear cell^a^	N/a	M	68
			Clear cell	Bilateral (n = N/a)	M	25
			Clear cell	Bilateral (n = N/a)	M	66
			Clear cell	Bilateral (n = N/a)	M	82
			Clear cell	Bilateral (n = N/a)	M	44
			Clear cell	Bilateral (n = N/a)	F	39
			Clear cell	Unilateral (n = N/a)	F	44
Bonne et al^18^	t(3;15)(p11;q21)	N/a	Clear cell	N/a	F	49
	ins(3;13)(p24.2;q32q21.2)		Clear cell	N/a	N/a	74
Foster et al^23^	t(3;6)(q22;q16.2)	N/a	Clear cell Papillary	Bilateral (n = 3)	M	49
Poland et al^24^	t(3;8)(p14;q24.1)		Clear cell	Bilateral (n = N/a)	F	47
			Clear cell	Bilateral (n = N/a)	M	39
Kuiper et al^25^	t(3;4)(q21;q31)	chr3:127177526 chr4:152360211	Clear cell	N/a	N/a	45
McKay et al^26^	t(2;3)(q37.3;q13.2)	N/a	Clear cell	Bilateral (n = 8)	M	54
			Clear cell	N/a	M	50
			Clear cell	Unilateral (n > 1)	F	35
Doyen et al*27*	t(11;22)(q23‐24;q11.2–12)	N/a	Clear cell	Unilateral (n = 1)	M	72
Wake et al^28^	t(5;19)(p15.3;q12)	chr5:6456877‐6 456 885 chr19:29788529‐29 788 531	Oncocytoma Chromophobe	Unilateral (n = 2)	F	35
			Clear cell Chromophobe Oncocytoma	Bilateral (n > 2)	F	36

Abbreviation: RCC, renal cell carcinoma.

a Individuals were presumed to be carriers of the relevant rearrangement but were not tested.

Assessment and characterization of further families and individuals carrying translocations associated with predisposition to RCC may help elucidate the genetic features and mechanisms that lead to disease onset in these patients. Here, we report the results of performing genome sequencing (GS) to characterize five constitutional rearrangements detected in individuals with RCC and interpret the results in the context of previous reports of RCC‐associated constitutional translocations.

## 2 MATERIALS AND METHODS

### 2.1 Literature review

Reports of cases of RCC with a constitutional chromosome rearrangement were identified through a search of PubMed using the search terms “renal cell carcinoma” or “renal cancer” or “kidney cancer/tumor” and “rearrangement/inversion/translocation or chromosome” and by searching of previously published reports (performed January 2019). When previous reports had suggested candidate genes that were either close to or disrupted by the relevant chromosomal breakpoints, evidence to suggest that the genes were implicated in human cancer was sought by reviewing curated data from the Network of Cancer Genes data portal (NCG; http://ncg.kcl.ac.uk/ version 6)^31^ (performed January 2019) where genes were classified as either “known cancer genes,” “candidate cancer genes,” or “non‐cancer genes.” Genes flagged as “false positive cancer genes” were designated as “non‐cancer genes.”

### 2.2 Clinical studies

Individuals presenting with RCC and with constitutional rearrangements were ascertained through Regional Clinical Genetics Units in the United Kingdom. DNA was extracted from whole blood according to standard protocol in the referring genetics service and, when available, paraffin embedded tumor material was obtained from the relevant hospital histopathology department. All patients gave written informed consent and the study was approved by the South Birmingham Ethics Committee.

### 2.3 Sequence alignment and variant calling

DNA from four probands was sequenced at Novogene. A total of >1 μg gDNA (1.2‐1.7 μg) at approximately 100 ng/μL was used for GS (×30 coverage). Generated FASTQ files were aligned to GRCh38 using BWA mem (version 0.7.15‐r1140).^32^ BAM files were sorted, polymerase chain reaction (PCR) duplicates removed, and indexed, after which Indel realignment and base score recalibration was performed using GATK IndelRealigner and BaseRecalibrator (version 3.7‐0‐gcfedb67),^33^ respectively. Genome‐wide variant calling was jointly performed on all samples using GATK unified genotyper (version 3.7‐0‐gcfedb67).^33^ DNA from one proband underwent GS as part of the NIHR BioResource Rare Diseases study with sequencing and primary bioinformatics performed as previously described.^34^ Data were aligned to genome build GRCh37 and all analyses were performed identically with appropriate adjustments for differences in genome build. All genomic coordinates are reported in GRCh38 and GRCh37 coordinates were remapped using the NCBI remap tool (https://www.ncbi.nlm.nih.gov/genome/tools/remap). Called SNVs were processed and filtered for various quality control metrics and allelic frequency (Table S1).

### 2.4 Genome sequencing analysis: Candidate gene analysis and breakpoint identification

The GS results were analyzed for evidence for rare, potentially pathogenic, SNVs, and copy number abnormalities in previously reported inherited RCC genes (*VHL*, *MET*, *FH*, *SDHB*, *SDHD*, *SDHC*, *BAP1*, and *CDKN2B*).^2^, ^4^ Copy number detection was performed using Canvas Copy Number Variant Caller (version 1.39.0.1598),^35^ copy number variants were filtered to include calls only marked as “PASS.” Structural rearrangements and breakpoints were identified using Manta Structural Variant Caller (version 1.3.1),^36^ Manta structural variants were filtered to include only calls marked as “PASS,” number of supporting spanning/split reads >5, QUAL >100, and call frequency (Table S3). Full details of bioinformatic processes are described in the Supporting Information. Breakpoints called on chromosomes matching cytogenetic reports were visually inspected using Integrative Genomics Viewer (IGV‐version 2.3.93) to confirm the presence of split and spanning reads (Figures S1‐S5). The data that support the findings of this study are available from the corresponding author upon reasonable request.

### 2.5 Topologically associated domain analysis

Topologically associated domains (TADs) reported by Dixon et al^37^ derived from human embryonic stem cells (GRCh38) were used as the reference TAD set at 40 kb resolution. Structural variation coordinates were intersected with TAD coordinates using bedtools (version 2.25.0).^38^ The corresponding TADs were then intersected with the genomic positions of all known gene loci^39^ (Supporting Information) to find genes contained within a given TAD and only protein‐coding genes were included. Protein‐coding genes identified within a TAD were assessed for potential function in cancer using the Network of Cancer Genes data portal (NCG; http://ncg.kcl.ac.uk/ version 6),^31^ as previously described. TAD regions were visualized using the Hi‐C data browser (http://promoter.bx.psu.edu/hi-c/index.html).^40^

### 2.6 Sanger sequencing

Direct sequencing of breakpoints was performed by Sanger sequencing using breakpoint spanning primer pairs (Table S2). PCR products were generated using Amplitaq Gold polymerase (Applied Biosystems, California) following the manufacturer's protocol. PCR products were sequenced using the BigDye Terminator v3.1 Cycle Sequencing Kit (Applied Biosystems) following the manufacturer's protocol. Termination sequencing products were purified by isopropanol precipitation, resuspended in Hi‐Di Formamide (Applied Biosystems), and sequenced on the ABI 3730 sequencing platform (Applied Biosystems). Sequences were aligned and analysed using Sequencher DNA analysis software (version 5.3.4; Gene Codes, Michigan).

### 2.7 Statistical tests

All statistical tests were performed using R project for statistical computing (version 3.5.1). Welch's *t* test was performed using the package BSDA (version 1.2.0) with the function tsum.test. Kruskal‐Wallis rank sum test was performed using the base R function kruskal.test. Fisher's exact test was performed using the base R function fisher.test. Statistical testing was undertaken on data from confirmed translocation carriers only.

## 3 RESULTS

### 3.1 Literature review of previously reported cases

**Figure 1:**
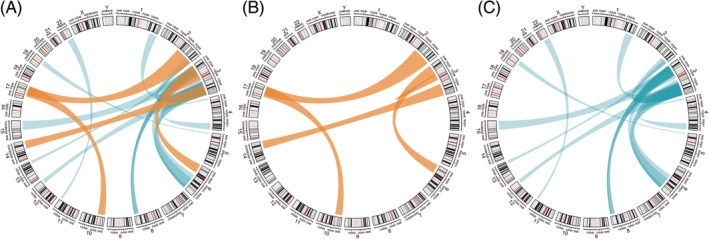
Circos plots visualizing constitutional chromosomal rearrangements. Previously published translocations are shown in blue and rearrangements identified in this study in orange. The width of the region at the ends of each ribbon represents the proportion of each chromosome which is translocated with its corresponding translocation partner. A, Contains all previously published translocations and translocations in the current series. B, Contains only previously published translocations. C, Contains only rearrangements in this series [Color figure can be viewed at http://wileyonlinelibrary.com]

**Figure 2:**
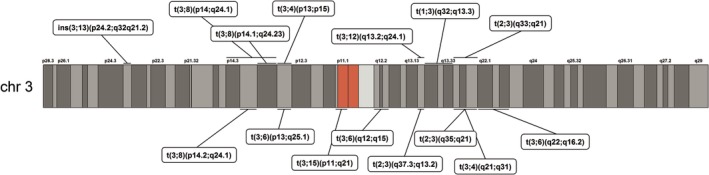
Diagram illustrating the position of chromosome 3 translocation breakpoints across the p and q arms. Differentially shaded portions represent different cytobands, the red region represents the centromeric region. Positions given in cases without base pair resolution are the median position for a given cytoband in the translocation karyotype [Color figure can be viewed at http://wileyonlinelibrary.com]

A total of 17 previously published distinct constitutional chromosome rearrangements were identified from searches of the biomedical literature (Table 1). In 15 cases (88%), chromosome 3 was involved (all of which were reciprocal translocations) and there were a variety of partner chromosomes in the 15 translocation cases (eg, three with chromosome 6, three with chromosome 8—Table 1 and Figure 1). For the RCC‐associated chromosome 3 translocation cases, the breakpoints were almost evenly distributed between the long arm (3q, n = 8) and short arm (3p; n = 7) and were heterogeneous (Figure 2).

Review of the clinical and pathological data in the previously reported cases demonstrated nine kindreds with at least two related individuals with RCC. In the four cases without a family history and available clinical information, multiple RCCs were described in two individuals. The mean age at diagnosis of a renal tumor in those cases known to carry a constitutional chromosomal rearrangement was 50 years (range 25‐82 years). Histopathological details were available for 43 cases and clear cell RCC was reported in 42 (98%) cases.

Previous studies have demonstrated that cases of sporadic and familial RCC differ by mean age of diagnosis, with RCC presenting earlier in familial cases.^41^, ^42^ Comparison of the mean age of diagnosis of RCC in translocation cases to familial and sporadic RCC cases (as reported previously by Maher et al^41^ and Woodward et al^42^) were 50.2 (SD = 12.7), 48.2 (SD = 12.3), and 61.8 (SD = 10.8) years of age, respectively. Translocation cases have a statistically lower age of diagnosis than those with sporadic disease (Welch's *t* test, *P* = 9.84 x 10^−7^) but no significant difference between translocation and familial cases was observed (Welch's *t* test, *P* = .522). Although age of diagnosis across all affected translocation carriers is variable, there was no significant difference in age between familial (with two or more related individuals) translocation cases (Kruskal‐Wallis test, *P* = .174).

The chromosomal rearrangement breakpoints had been mapped in 15 of 17 previously reported cases and a total of 10 candidate genes had been reported to be disrupted by the relevant rearrangement breakpoints (Table 2). Additionally, 21 genes found to be in the vicinity of translocation breakpoints and cited as relevant genes by the authors of the original report were also assessed (Table 3). The evidence for implicating the various genes in RCC predisposition was assessed using NCG data portal (Tables 2 and 3). Of the 10 genes directly disrupted by translocation breakpoints, two are classified as known cancer genes, with all remaining genes having no evidence supporting their role in cancer. With regards to 21 genes stated to be in the vicinity of a translocation breakpoint, two were designated as known cancer genes and four were classified as candidate cancer genes.

**Table 2:** Reassessment of genes disrupted by translocation breakpoints in RCC‐associated translocations reported previously

Original publication	Affected genes	Position (GRCh38)	Known cancer gene (NCG 6.0)
Cohen et al^5^	*FHIT*	chr3:59747587‐61 251 459	Known cancer gene
Cohen et al^5^	*RNF139 (TRC8)*	chr8:124474738‐124 488 618	Non‐cancer gene
Kovacs and colleagues^16^	*STXBP5*	chr6:147204358‐147 390 476	Non‐cancer gene
Koolen et al^17^	*SLC49A4 (DIRC2)*	chr3:122794795‐122 881 139	Non‐cancer gene
van Kessel et al 18	*KCNIP4*	chr4:20728606‐21 948 801	Non‐cancer gene
Kanayama et al 20	*LSAMP*	chr3:115802363‐117 139 389	Non‐cancer gene
Kanayama et al 20	*RASSF5 (NORE1)*	chr1:206507530‐206 589 448	Non‐cancer gene
Podolski et al 21	*DIRC1*	chr2:188733738‐188 839 420	Non‐cancer gene
Kuiper et al^25^	*FBXW7*	chr4:152320544‐152 536 095	Known cancer gene
Wake et al^28^	*UBE2QL1*	chr5:6437347‐6 496 721	Non‐cancer gene

*Note*: Genes were categorized according to their current status in NCG v6.0 (Repana et al^31^).

Abbreviations: NCG, network of cancer genes; RCC, renal cell carcinoma.

**Table 3:** Reassessment of genes highlighted as being close to translocation breakpoints in RCC‐associated translocations reported previously

Original publication	Affected genes	Position (GRCh38)	Known cancer gene (NCG 6.0)
Meléndez et al^22^	*LRIG1*	chr3:66378797‐66 501 263	Candidate cancer gene
Wake et al^28^	*CCNE1*	chr19:29811898‐29 824 312	Known cancer gene
Kuiper et al^25^	*C3orf56*	chr3:127193131‐127 198 185	Non‐cancer gene
Foster et al^23^	*PPP2R3A*	chr3:135965673‐136 147 891	Non‐cancer gene
Foster et al 23	*PCCB*	chr3:136250306‐136 337 896	Non‐cancer gene
Foster et al 23	*STAG1*	chr3:136336233‐136 752 403	Known cancer gene
Foster et al 23	*MSL2 (RNF184)*	chr3:136148922‐136 197 241	Non‐cancer gene
Foster et al 23	*EPHB1*	chr3:134597801‐135 260 467	Non‐cancer gene
Foster et al 23	*EPHA7*	chr6:93240020‐93 419 547	Non‐cancer gene
Podolski et al^21^	*HIBCH*	chr2:190189735‐190 344 193	Non‐cancer gene
Podolski et al 21	*INPP1*	chr2:190343470‐190 371 665	Non‐cancer gene
Podolski et al 21	*HNRNPC* (*HNRPC*)	chr14:21209136‐21 269 494	Non‐cancer gene
Koolen et al 17	*HSPBAP1*	chr3:122740003‐122 793 824	Non‐cancer gene
Koolen et al 17	*SEMA5B*	chr3:122909082‐123 028 605	Candidate cancer gene
Kovacs et al 16	*PDZRN3*	chr3:73382433‐73 624 940	Candidate cancer gene
Kovacs et al 16	*CNTN3*	chr3:74262568‐74 521 140	Non‐cancer gene
Kovacs et al 16	*NECTIN3* (*PVRL3*)	chr3:111070071‐111 275 563	Non‐cancer gene
Kovacs et al 16	*HSPB8*	chr12:119178642‐119 221 131	Candidate cancer gene
Kovacs et al 16	*CCDC60*	chr12:119334712‐119 541 047	Non‐cancer gene
Cohen et al^5^	*TRMT12*	chr8:124450820‐124 462 150	Non‐cancer gene
Cohen et al^5^	*TATDN1*	chr8:124488485‐124 539 458	Non‐cancer gene

*Note*: Genes were categorized according to their current status in NCG v6.0 (Repana et al*31*).

Abbreviations: NCG, network of cancer genes; RCC, renal cell carcinoma.

### 3.2 Clinical features of previously unreported cases

Five previously unreported constitutional chromosomal rearrangements ascertained through a patient presenting with RCC were identified through UK genetics services. The cytogenetic, clinical features, and pathological features of the five probands and (where relevant) their affected relatives are described in Table 4. There were four translocations (involving chromosome 3 in two cases) and a pericentric inversion of chromosome 3 (Table 4 and Figure 1). Two or more individuals developed RCC in three kindreds.

**Table 4:** Clinical details of families harboring RCC‐related translocations cases in this series

Chromosomal alteration	Individual	Carrier	Sex	Age	Histology (RCC)	Type(foci = n)	Sanger	Breakpoints	Additional notes
t(2;17)(q21.1;q11.2)	Proband	Yes	M	37	Clear cell	N/a	Yes	chr2:130693727 chr17:28031855	Some areas of spindle cell changes
	Paternal grandfather	Unknown	M	N/a	Not specified	N/a		N/a	N/a
	Mother	Yes	F	58	Unaffected	N/a		N/a	N/a
	Sibling 1	Yes	?	40	Unaffected	N/a		N/a	N/a
	Sibling 2	Yes	?	31	Unaffected	N/a		N/a	N/a
t(3;6)(p14.2;p12)	Proband	Yes	M	72	Not specified	N/a	Yes	chr3:66680663 chr6:54817716	N/a
	Relative 1	Yes	?	55	Clear cell	Bilateral (n = N/a)	No		Unilateral recurrent RCC and an adrenal metastasis, age 74 years
	Relative 2	Yes	?	N/a	Not specified				
	Relative 3	Yes	?	N/a	Unaffected				Last follow up 47‐52 years
	Relative 4	Yes	?	N/a	Unaffected				Last follow up 47‐52 years
	Relative 5	Yes	?	N/a	Unaffected				Last follow up 47‐52 years
inv(3)(p14.2q12)	Proband	Yes	F	N/a	Unaffected	N/a	No	chr3:59964935 chr3:98667603	
	Cousin	Yes	M	39	Clear cell	N/a			
	Paternal aunt	Yes	F	N/a	Unaffected				
	Father	Yes	M	N/a	Unaffected				
	Grandfather	Yes	M	N/a	Unaffected				Carcinomatosis aged 80 years
	Brother	N/a	M	48	Not specified	N/a			
t(3;14)(q13.3;q23)	Proband	Yes	M	75	Clear cell	n = 2	Yes	chr3:125771297 chr14:59009871	Bladder carcinoma age 77 years
	Nephew	Yes	M	67	Not specified	N/a	Yes	chr3:125771297 chr14:59009871	
	Brother	Obligate	M	79	Not specified	N/a	No	N/a	
	Daughter	Unknown	F	36	Not specified	N/a	No	N/a	
	Brother	Unknown	M	41	Not specified	N/a	No	N/a	
	Mother	Unknown	F	50	Not specified	N/a	No	N/a	
t(10;17)(q11.21;p11.2)	Proband	Yes	M	53	Clear cell	N/a	Yes	N/a	Fibrofolliculomas, pneumothoraces
	Relative	Yes	F	N/a	Unaffected	N/a		N/a	Fibrofolliculomas, multiple lung and renal cysts

Abbreviation: RCC, renal cell carcinoma.

In the kindred with the t(3;14)(q13.3;q22), six individuals developed RCC (three of whom were confirmed or obligate translocation carriers). The proband presented with bilateral clear cell RCC at age 75 years, his daughter died from RCC at age 36 years, his mother and two of his brothers were reported to have developed RCC at ages 51, 41, and 79 years, respectively. The proband's brother was an obligate t(3;14)(q13.3;q22) carrier and his son developed RCC at age 67 years and was confirmed to be a translocation carrier.

In the kindred with the t(3;6)(p14.2;p12) rearrangement, the proband presented with RCC at age 72 years and four relatives were demonstrated to also harbor the translocation. Three had not developed RCC (age at last follow up 47‐52 years) but one (the proband's brother) had developed bilateral clear cell RCC at age 55 years with unilateral recurrent disease and an adrenal metastasis at age 74 years and his son died from RCC at age 40 years without any record of his status for the t(3;6)(p14.2;p12) translocation.

The index case in whom the inv(3)(p21.1q12) was identified was unaffected but was ascertained following a report that her cousin had developed clear cell RCC at age 39 and harbored the chromosome 3 inversion. Other unaffected carriers of the inversion in the family included her paternal aunt and father, while her grandfather was also to be a carrier and died of carcinomatosis around age 80 years. The proband's brother was diagnosed with RCC at age 48 but was not tested for the inversion.

The t(2;17)(q21;q11.2) was identified in a 37‐year‐old man with a poorly differentiated in part clear cell RCC who died from metastatic disease shortly thereafter. The translocation was maternally inherited and was detected in three unaffected family members (mother and two siblings) aged between 30 and 58 years of age.

In the kindred with the t(10;17)(q11.22;p12) the proband, with his sister, were found to have features of suggestive Birt‐Hogg‐Dubé syndrome (BHD; MIM 135150; pneumothoraces, and fibrofolliculomas in the proband and multiple pulmonary cysts and fibrofolliculomas in the sister) after the diagnosis of RCC in the proband and the detection of the translocation.

### 3.3 Molecular characterization of constitutional rearrangements in previously unreported cases

GS did not identify any plausible likely pathogenic or pathogenic SNVs or CNVs in previously reported inherited RCC genes (V*HL*, *SDHB*, *SDHC*, *SDHD*, *MET*, *FLCN*, *FH*, *BAP1*, and *CDKN2B*) in the four probands who were affected by RCC (the index case with the inv(3)(p21.1q12) had a family history of RCC but had not developed RCC). A novel missense variant of uncertain significance by ACMG criteria^43^ was identified in *PBRM1* (NM_018313.4:c.2446A>T p.Asn816Tyr) in the t(3;6)(p14.2;p12) case. DNA from an affected individual was not available for sequencing in the family carrying the inv(3)(p21.1q12), as such sequencing was performed solely to identify candidate breakpoints. Candidate rearrangement breakpoints were identified from the GS data by the Manta structural variation detection algorithm in all five cases.

Breakpoints for translocation t(3;14)(q13.3;q22) were resolved to be present at the loci chr3:125771297 and chr14:59009871‐59 009 875. The candidate breakpoints were supported by 7 and 9 spanning and split reads, respectively (Table S3). The candidate breakpoint locations identified by GS differed from those suggested previously by cytogenetic studies. The 3q breakpoint at chr3:125771297 is within cytoband 3q21 and the GS‐identified 14q breakpoint at chr14:59009871 maps to 14q23 with respective genomic distances of 7.3 and 4.7 Mb from the reported cytogenetic bands seen by karyotyping. Sanger sequencing confirmed the presence of the translocation breakpoints. Sanger sequencing in a DNA sample from his affected nephew confirmed identical breakpoints to the proband. The 3q breakpoint intersects with *LOC105374312*, an uncharacterized noncoding RNA gene and the 14q breakpoint disrupts the last intron of *LINC01500*, a long intergenic noncoding RNA gene, and is predicted to result in a truncated transcript lacking the final exon.

GS in the second chromosome 3‐associated translocation case t(3;6)(p14.2;p12) revealed candidate breakpoints at chr3:66680663 and chr6:54817716 within an AT‐rich repetitive region. Breakpoint calls were supported by 4 and 7 spanning and split read calls, respectively (Table S3). Discordance between karyotyping and GS‐derived cytoband positions was limited to adjacent bands with 3p14.2 being mapped to 3p14.1 (5.5 Mb centromeric) and 6p12 being defined at a greater resolution at 6p12.1. Sanger sequencing confirmed the presence of the translocation breakpoints. The 3p chromosomal breakpoint identified by GS mentioned above disrupted LOC105377142, an uncharacterized noncoding RNA. The 6p breakpoint did not disrupt a predicted gene but was 29 kb upstream of *FAM83B* in 6p12.1.

The candidate breakpoints in the inv(3)(p21.1q12) were identified by Manta with 11 spanning and 11 split reads supporting the presence of this inversion, though the number of reference spanning reads was only 2 (Table S3). The two candidate breakpoints mapped to chr3:59964935 at 3p14.2 (interrupting intron 7 of *FHIT*) and chr3:98667603 at 3q12 (47 kb upstream of *ST3GAL6‐AS1*, a noncoding RNA gene). The discrepancy between the cytogenetic position and GS‐derived positions did not greatly deviate from other differences seen in other cases with the 3p breakpoint at 3p21 detected 6.6 Mb closer to the centromere at 3p14.2. Although cytogenetics and Manta calls support the presence of the inv(3)(p14.2q12), Sanger sequencing under multiple experimental conditions failed to generate any PCR products and the candidate breakpoints could not be independently confirmed.

Assessment of the DNA at the described breakpoints for the inv(3)p14.2q12) rearrangement was performed to determine if local DNA features and nucleotide composition may explain the failure to confirm the inversion by Sanger sequencing. Analysis of each breakpoint within a ±1 Kb window demonstrated a lower than average GC‐content percentage at both sites (chr3:59963935‐59 965 935 = 32.3% and chr3:98666603‐98 668 603 = 36.6%) compared to genome‐wide GC content. Furthermore, the 3p14.2 breakpoint occurred within proximity of two repeat elements (chr3:59965304‐59 965 360‐(AT)n and chr3:59965818‐59 965 936‐L3) and the 3q12 breakpoint overlapped with a repetitive region (chr3:98667322‐98 667 927‐L1M2), as well as in proximity of five further repetitive DNA elements, as defined by RepeatMasker. Taken together, particularly when considering the calling of multiple breakpoints by Manta, low complexity and additional undetermined structural variation at either one or both breakpoints may explain the failure to confirm the breakpoints by Sanger sequencing.

GS in the first of the two non‐chromosome 3 translocations t(2;17)(q21;q11.2) localized the breakpoints to chr2:130693728 (2q21.1) and chr17:28030855 (17q11.2). The translocation breakpoint was supported by 9 spanning and 10 split reads as called by Manta (Table S3). Sanger sequencing confirmed the genomic coordinates and breakpoint as a single base translocation without local rearrangement, insertions, or deletions. Cytogenetic positions were inconsistent for chromosome 2 (q21) with the next generation sequencing (NGS) breakpoint occurring in the adjacent band q21.1, proximately 5.3 Mb closer to q telomere. The breakpoint present on chromosome 2 disrupted the coding region of two overlapping pseudogenes *KLF2P3* and *FAR2P3*, as well as interrupting a CpG island spanning chr2:130693485‐130 693 839. The nearest coding genes were *POTEJ*, *AMER3*, and *GPR148* which were 35 kb upstream, 34 kb downstream, and 62 kb downstream, respectively. The junction on chromosome 17 did not disrupt any known coding region but was 1.7 kb upstream of a reported H3K27Ac element spanning chr17:28033593‐28 035 092, and 9.9 kb upstream of the *NLK* gene.

The second non‐chromosome 3 translocation t(10;17)(q11.22;p12) underwent sequencing as part of the NIHR BioResource Rare Diseases project (see methods) and was analyzed previously as part of a multiple primary tumor cohort 34 with a history of facial fibrofolliculomas, recurrent pneumothoraces, and RRC. At that time, no abnormality was detected but subsequently reanalysis identified candidate translocation breakpoints that were supported by two overlapping Manta calls for the chromosome 10 and chromosome 17 breakpoints at chr17:17218211‐17 218 214 (17p11.2) and chr10:43236047‐43 236 050 *(*10q11.21) that were supported by 22 spanning and 10 split reads and a secondary call at chr17:17218216‐17 218 217 and chr10:43236058‐43 236 059 by 15 spanning and 18 split reads (Table S3). As with other cases, differences between breakpoints on chromosome 10 and 17 from both karyotyping and GS were found with 10q11.22 mapping to 10q11.21 (3.3 Mb centromeric) and 17p12 mapping to 17p11.2 (3.7 Mb centromeric). Given the proximity of the assigned breakpoint regions, a single translocation was presumed with an additional nested structural variation resulting in divided calling. Sanger sequencing confirmed the presence of the translocation breakpoint in the proband. The chromosome 17 breakpoint prediction disrupted the coding region of *FLCN*, falling within intron 9 (ENST00000285071). The chromosome 10 breakpoint disrupted the first intron of *RASGEF1A* (the first exon encodes the 5′ untranslated region proximal to the translation initiation site (ENST00000395810). The proband's sibling, who was known to carry the t(10;17)(q11.22;p12), was also found to have evidence of BHD syndrome (multiple lung and renal cysts and facial fibrofolliculomas).

Although RNA was not available from the t(10;17)(q11.21;p11.2) proband to assess fusion gene formation, both genes are on the negative strand and do not appear to interrupt splice site consensus sequences, suggesting fusion gene products could be transcribed consisting of exon 1 of *RASGEF1A* with exons 10 to 14 of *FLCN* and exons 1 to 9 of *FLCN* with exons 2 to 13 of *RASGEF1A*, from each derivative chromosome, respectively. Translocations as determined by karyotyping, NGS cytobands, standardized nomenclature, and cytoband discrepancies are noted in Table 5. Translocations will be referred to by the shortened nomenclature system as described by Ordulu et al^44^ in both the text and tables hereafter.

**Table 5:** Details of karyotype, NGS‐derived cytogenetic positions, and standardized nomenclature for chromosomal alterations described in this series

Karyotype	NGS—short nomenclature	NGS—detailed nomenclature
t(3;14)(q13.3;q22)	seq[GRCh38/hg38]t(3;14)(q21;q23)	seq[GRCh38/hg38]t(3;14) (3pter➔3q21(125771297)::14q23(59 009 871~59 009 875)➔14qter; 14pter➔14q23(59 009 871~59 009 875)::3q21(125771298)➔3qter)
t(3;6)(p14.2;p12)	seq[GRCh38/hg38]t(3;6)(p14.1;p12.1)	seq[GRCh38/hg38]t(3;6) (3pter➔p14.1(66680663)::6p12.1(54817717)➔6qter; 6pter➔6p12.1(54817716)::3p14.1(66680664)➔3qter)
inv(3)(p21.1q12)	seq[GRCh38/hg38]inv(3)(p14.2q12)	seq[GRCh38/hg38]inv(3) (qter➔q12(98667604)::p14.2(59964936‐98 667 603)::p14.2(59964935)➔pter)
t(2;17)(q21;q11.2)	seq[GRCh38/hg38]t(2;17)(q21.1;q11.2)	seq[GRCh38/hg38]t(2;17) (2pter➔2q21.1(130693728)::17q11.2(28030856)➔17qter; 17pter➔17q11.2(28030855)::2q21.1(130693729)➔2qter)
t(10;17)(q11.22;p12)	seq[GRCh38/hg38]t(10;17)(q11.21;p11.2)	seq[GRCh38/hg38]t(10;17) (10pter➔10q11.21(43 731 495~43 731 506)::17q11.2(17 121 528~ 17 121 531)➔17qter; 17pter➔17q11.2(17 121 525~ 17 121 530)::10q11.21(43 731 498~43 731 507)➔10qter)

Abbreviation: NGS, next generation sequencing.

### 3.4 Computational evaluation of breakpoint‐related genes

The five constitutional rearrangements were confirmed or postulated to disrupt three protein coding genes (*FHIT*, *FLCN*, and *RASGEF1A*). Two of these genes, *FHIT* and *FLCN*, have been previously implicated as renal TSGs^45^, ^46^ and the NCG data portal classified both *FHIT* and *FLCN* as “known cancer genes,” *RASGEF1A* as a “candidate cancer gene.”

Assessment of the effect of a translocation on the surrounding genomic architecture and consequently the impact on gene regulation is more challenging. Within the nucleus, DNA is rearranged into complex two dimensional and three‐dimensional structures and this spatial organization directly impacts biological function. Higher order chromatin structures such as TADs have been identified as pervasive and highly conserved features of genome organization^47^ and disruption of these TADs and their associated genomic boundaries can lead to gene dysfunction, ectopic genomic interactions, and disease phenotypes.^48^, ^49^ We sort to assess if any breakpoints occurred within TADs and to what extent, if any, these disruptions could dysregulate long range gene regulatory structures.

A total of 8/10 rearrangement breakpoints occurred within a TAD (or a TAD boundary region), with the chromosome 3 breakpoint in t(3;14)(q21;q23) and chromosome 2 breakpoint in t(2;17)(q21.1;q11.2) occurring within “unorganized chromatin” regions (Table 6 and Figures S7‐S16). TADs which harbored a breakpoint were assessed for encapsulated genes and the subsequently identified genes assessed for relevance to cancer via the NCG (Table 6). Analysis demonstrated two known cancer genes (*NCOA4* and *RET*) and a further five candidate cancer genes (*LLGL1*, *LRIG1*, *LYRM9*, *ST3GAL6*, and *TMEM199*) were within breakpoint‐containing TADs.

**Table 6:** Assessment of genes disrupted by (*) or within the same topologically associating domain as RCC‐associated rearrangement reported in the current series

Chromosomal alteration	Chr.	Start	End	TAD chr.	TAD start	TAD end	Cancer genes
inv(3)(p21.1q12)	chr3	59 964 935	59 964 935	chr3	59 920 000	61 400 000	FHIT* a
	chr3	98 667 603	98 667 603	chr3	98 600 000	99 800 000	*ST3GAL6* b
t(10;17)(q11.22;p12)	chr17	17 218 211	17 218 214	chr17	16 840 000	18 400 000	FLCN* a *LLGL1* b
	chr10	43 236 047	43 236 050	chr10	41 680 000	46 360 000	*NCOA4* a RASGEF1A* b *RET* a
t(2;17)(q21.1;q11.2)	chr2	130 693 728	130 693 728	NA	N/A	N/A	N/A
	chr17	28 030 855	28 030 855	chr17	27 640 000	28 360 000	*LYRM9* b *TMEM199* b
t(3;14)(q13.3;q22)	chr3	125 771 297	125 771 297	NA	N/A	N/A	N/A
	chr14	59 009 871	59 009 871	chr14	58 440 000	59 080 000	N/A
t(3;6)(p14.2;p12)	chr6	54 817 716	54 817 716	chr6	53 720 000	55 240 000	N/A
	chr3	66 680 663	66 680 663	chr3	66 240 000	66 880 000	*LRIG1* b

*Note*: Genes were categorized according to their current status in NCG v6.0 (Repana et al^31^).

Abbreviation: RCC, renal cell carcinomal; TAD, topologically associated domain.

a Known cancer gene.

b Candidate cancer gene.

### 3.5 Tumor analysis

Tumor material was available from an affected individual with the familial t(3;14)(q21;q23), and expert histopathological review classified the two separate tumors (*A* = 3 cm and *B* = 3.8 cm) for which material was received as clear cell RCC. Tumor A was classed as WHO/ISUP Grade 2 B (as focal grade but predominantly grade 2). No necrosis was seen. Immunohistochemistry on sections from Tumor B demonstrated staining of moderate intensity with CA‐IX and AE1/3 and weak focal staining with vimentin. Only rare cells were CK7 positive and the tumor was negative for CD117, HMB45, and Mel‐A, an immunoprofile consistent with the diagnosis.

Using an NGS sequencing panel of 68 cancer‐related genes, as described previously,^50^ tumor sequencing was performed on the two renal tumors to assess *VHL* mutation state. The larger tumor harbored a frameshift deletion in *VHL* (NM_000551: c.408delT: p.Phe136Leufs*23: rs397516442) in 49% of reads but no somatic mutations in *VHL* were detected in the smaller tumor. Analysis of the other genes on the sequencing panel did not demonstrate any protein‐affecting somatic alteration either tumor at a variant allele fraction greater than 10%.

Sufficient DNA was available for one of the t(3;14)(q21;q23) tumors (tumor B, 3.8 cm diameter) to perform genome‐wide copy number assessment using the Applied Biosystems OncoScan CNV FFPE assay kit as described previously.^51^ The OncoScan assay identified chromosomal alterations consistent with the loss of the der (3), including 3p, and retention of the wild type chromosomes (3 and 14) and der (14) (arr[GRCh37] 3p26.3q21.2(63410‐125495356)x1,(5)x3,14q23.1q32.33(59491095‐107282024)x1,(X)x1). Additionally, the tumor also harbored trisomy 5 and loss of chromosome Y (Supporting Information and Figures S17 and S18).

## 4 DISCUSSION

We describe five previously unreported RCC‐associated constitutional chromosomal rearrangements that increase the total number of rearrangements reported to 22 and the number of cases in which the breakpoints have been characterized to 20. We found that GS enabled both the identification of candidate translocation breakpoints and simultaneously excluded coincidental pathogenic SNVs and CNVs in known hereditary cancer genes. With the increasing availability and decreasing cost of GS, it will become increasingly feasible to characterize the molecular pathology of RCC‐associated constitutional chromosomal rearrangements. This will improve our understanding of the relevance of individual RCC‐associated constitutional chromosomal rearrangements to the RCC tumorigenesis and we found that the breakpoint location reported on routine cytogenetic analysis often did not correspond to the breakpoint locations identified by GS. The majority (21/22, 95.5%) of RCC‐associated constitutional chromosomal rearrangements reported to date have been associated with the clear cell variant of RCC. This is the most common histological subtype of sporadic RCC (75‐80%) and is characterized by somatic inactivation of *VHL* and deletions of the short arm of chromosome 3.^8^, ^9^, ^15^, ^52^ The mean age at diagnosis of RCC in the cases reported to date (51 years, range 25‐82, n = 57, SD = 13.25) is younger than the average age for sporadic RCC (eg, 61.8 years).^41^ While this is a feature of other forms of hereditary RCC (and many other inherited cancer types), there may also be an element of ascertainment bias with early onset cases more likely to be investigated for a genetic cause. In the largest family we identified, t(3;14)(q21;q23), the mean age at diagnosis of RCC in the six affected cases was 58 years and three individuals were either known or obligate translocation carriers. Although the breakpoints characterized by this translocation do not disrupt any known cancer gene, given the loss of the derivative chromosomes is reported as the potential initiator of tumorigenesis in chromosome 3 translocations, the loss of der (3) would also result in the loss of 14q that would include the *HIF1A* coding region, which is a candidate 14q TSG.^53^

In both our own and the previously published literature series, most RCC‐associated constitutional chromosome rearrangements involved chromosome 3. Although this is consistent with the high frequency of 3p allele loss in sporadic clear cell RCC, the fundamental role of somatic inactivation of the *VHL* TSG in clear cell RCC and the incidence of somatic mutations of *PBRM1*, *BAP1*, and *SETD2* in RCC, to date most RCC‐associated constitutional chromosome 3 rearrangements do not appear to disrupt known RCC TSGs mapping to 3p. A potential explanation for this is the observation that RCC from individuals with a constitutional chromosome 3 translocation can show a somatic *VHL* mutation on the wild‐type chromosome 3 and loss of the derivative chromosome containing 3p (resulting in biallelic inactivation of the *VHL* TSG). This mechanism of tumorigenesis would imply that the susceptibility to RCC might result from instability of the translocated chromosome rather than disruption of a specific RCC TSG at the translocation breakpoint on chromosome 3^30^ and would be consistent with the variability of the RCC‐associated chromosome 3 rearrangement breakpoints described to date (Table 1). Indeed, analysis of the larger of the two t(3;14)(q21;q23)‐associated RCCs revealed a somatic truncating *VHL* mutation and copy number alterations consistent with loss the of der (3) translocated chromosome that included 3p as described previously.^30^ Nevertheless, it is interesting that the chromosome 3 inversion we described was associated with a breakpoint within *FHIT*. Previously, it was demonstrated in two apparently unrelated families with an RCC‐associated t(3;8)(p14.2;q24.1) which were reported to have harbored breakpoints that disrupted *FHIT* and *RNF139* (*TRC8)* on 3p and 8q, respectively.^22^, ^29^ *FHIT* is listed as a Tier 1 known cancer gene in the Cancer Gene Census (https://cancer.sanger.ac.uk/cosmic/census); however, the presence of a somatic VHL mutation and loss of the translocated chromosome 3 in a previous t(3;8)(p14.2;q24.1)‐associated RCC was unexpected.^5^, ^22^

It is possible that the recurrent involvement of *FHIT* in RCC‐associated chromosome 3 rearrangements reflects the presence of palindromic AT‐rich repeats at the t(3;8)(p14.2;q24.1) breakpoint and causes a propensity to recurrent rearrangements at this locus,^54^ although we note that only a fraction of chromosome 3 translocations are associated with predisposition to RCC.^55^ It is therefore conceivable that both instability of the translocated chromosome and monoallelic inactivation of *FHIT* contribute to RCC susceptibility. Analysis previously reported genes associated with translocations, as described by previous authors, and examination of TAD structures in current series identified several genes that have been previously reported to be located at or close to the breakpoints of RCC‐associated chromosome 3 rearrangements (Tables 2, 3, and 6). These genes were reviewed to determine which were included in recently compiled lists of known cancer genes which are based on the results of recent large‐scale cancer genomics projects; eight genes (*FHIT*, *LRIG1*, *FBXW7*, *CCNE1*, *STAG1*, *SEMA5B*, *PDZRN3*, and *HSPB8*) were identified as known or candidate cancer genes from previous publications. In addition, genes that were disrupted (*FHIT*, *FLCN*, *and RASGEF1A*) or occurred within a relevant TAD structure coinciding with the breakpoints of the novel RCC‐associated rearrangements reported here were also assessed. A total of 10 genes that were disrupted or within breakpoint‐associated TAD structures were classified as known (*FHIT*, *FLCN*, *NCOA4*, and *RET)* or candidate cancer genes (*LLGL1*, *LRIG1*, *LYRM9*, *RASGEF1A*, *ST3GAL6*, and *TMEM199*; Table 6). The independent occurrence of *LRIG1* in two separate cases (t(3;6)(p14.1;p12.1) in this series and in Meléndez et al^22^) is notable. Neither of the *LIRG1*‐related translocation breakpoints directly disrupted the coding region of *LRIG1* but occurred within the TAD (or in close proximity in the case of the latter) of the gene. *LRIG1* is known to encode a cell surface protein (leucine‐rich repeats and immunoglobulin‐like domains 1; LRIG1), which is known to negatively regulate epidermal growth factor receptor^56^ and ERBB‐family receptor degradation including RET and MET,^57^, ^58^ and deletions of *LRIG1* occur somatically in RCC cases at a rate of 2.3% within the TCGA dataset,^15^ of which 94% were clear cell RCC.

Relatively few RCC‐associated constitutional translocations not involving chromosome 3 have been reported. In addition to the two novel cases reported here, there are two previously reported cases^27^, ^28^ and the translocation breakpoints were characterized in only one of these cases. It is entirely possible that non‐chromosome 3 constitutional translocations and RCC might occur coincidentally and we note that, though there was an early age at onset (37 years) in the proband with t(2;17)(q21.1;q11.2) and an unconfirmed family history of RCC in his paternal grandfather, the translocation was also found in his mother and two siblings who were unaffected at ages 58, 40, and 31 years. However, identification of a translocation breakpoint that disrupted the *FLCN* gene in a patient with a t(10;17)(q11.21;p11.2) illustrated the value of characterizing all RCC‐associated constitutional rearrangements. Inactivating mutations in *FLCN* cause BHD syndrome which is characterized by facial fibrofolliculomas, pulmonary cysts, and pneumothorax and RCC.^45^, ^59^ The occurrence of fibrofolliculomas is age‐dependent and pneumothorax occurs in a minority of cases and so BHD may present with non‐syndromic RCC.^60^ However in the family reported herein, the t(10;17)(q11.21;p11.2) was associated with other evidence of BHD syndrome. To our knowledge, this is the first description of a constitutional translocation causing BHD syndrome.

The other novel translocation case did not disrupt a known cancer gene but occurred close to *NLK* (Nemo‐like kinase), a serine/threonine‐protein kinase, which has been associated with the noncanonical Wnt and MAPK signaling pathways. Although *NLK* is currently not designated as a known cancer gene, evidence of tumor suppressor activity has been reported^61^, ^62^, ^63^ and a role for NLK in the stabilization of TP53 has been suggested.^64^ Interestingly, NLK appears to collaborate with FBXW7 in the ubiquitination of MYB by enhancing ligation of additional ubiquitin molecules via NLK phosphorylation, leading to downregulation of cellular proliferation^65^ and, previously, an RCC‐associated constitutional translocation, t(3;4)(q21;q31), was demonstrated to interrupt *FBXW7*.^25^ Furthermore, *FBXW7* is a designated TSG, that is, mutated in multiple types of primary cancers and encodes an F‐box protein that is part of a SCF complex thought to target cyclin E and mTOR for ubiquitin‐mediated degradation.^66^, ^67^ Very recently, *FBXW7* has been identified as a novel cancer predisposition gene following an analysis of individuals with Wilms tumor.^68^ Additionally, it was demonstrated that FBXW7 interacts with ubiquitin‐conjugating enzyme E2Q‐like protein 1; this gene is known to be disrupted in a previously reported RCC translocation case,^28^ suggesting an interesting connection between multiple interacting gene products in translocation‐related RCC.

In conclusion, we report five new cases of RCC‐associated constitutional chromosome rearrangements characterized by GS. These include the first example of a chromosome 3 inversion associated with RCC, the first case of a major inherited RCC gene disrupted by a translocation and a third example of an RCC constitutional chromosome rearrangement that disrupts *FHIT*. Review of the five novel cases reported here and previously reported cases demonstrates that RCC‐associated constitutional chromosome rearrangements (a) mostly involve chromosome 3 but rearrangements that solely involve other chromosomes may also be causally linked to RCC, (b) may predispose to RCC by a variety of mechanisms including disruption of a TSG (eg, *FLCN*) and/or chromosomal instability (as with chromosome 3 translocations), (c) can be efficiently characterized by GS, and (d) can identify candidate pathways for RCC tumorigenesis. For chromosome 3 translocations, it is unclear why most cases that are not ascertained because of a personal or family history of RCC appear to be associated with a very low risk of RCC.^55^ In those translocations that do predispose to RCC, there may be a combination of factors involved including instability of the translocated chromosome during cell division together with disruption of a TSG (eg, *FHIT*) and/or polygenic effects that increase RCC susceptibility.

## Supporting information


**Appendix** **S1**: Supporting Information

## Data Availability

The data that support the findings of this study are available from the corresponding author upon reasonable request.
