# Supplementary material for: Characterization of renal cell carcinoma‐associated constitutional chromosome abnormalities by genome sequencing
Source: Genes Chromosomes Cancer. 2020 Feb 5;59(6):333–47. doi: 10.1002/gcc.22833 (PMC7187337; doi:10.1002/gcc.22833)

**Characterisation of renal cell carcinoma-associated constitutional chromosome abnormalities by whole genome sequencing – Supporting Information**

Bioinformatic scripts are provided here: <https://github.com/maherlab/RCC_translocation/>

**PRIMARY BIOINFOMATICS**

**Software and tools**

| **Process** | **Software (version)** |
| --- | --- |
| FASTQ alignment | BWA mem (version 0.7.15-r1140) |
| Remove PCR duplicates | samtools (version 1.6-12-gc7b2f4f) |
| Indel realignment | GATK IndelRealigner (version 3.7-0-gcfedb67) |
| Base score quality recalibration | GATK BaseRecalibrator (version 3.7-0-gcfedb67) |
| Sort & Index | samtools (version 1.6-12-gc7b2f4f) |
| Variant calling | GATK unified genotyper (version 3.7-0-gcfedb67) |

**SNV variant filtering**

Variant filtering for SNVs is performed by VCFtools (version 0.1.15) and an In-house bash and R scripts for various quality and filtering metrics. Variants were annotated using Annovar (release 2016-02-01) and pathogenicity established using the available databases, clinical information, and applying ACMG guidelines (Richards et al 2015).

**Table 1**

| **Minimum mean read depth** | **Minimum genotype quality** | **Maximum cohort minor allele frequency** | **Maximum missingness per site** | **Allele frequency in ExAC and 1000 Genomes** |
| --- | --- | --- | --- | --- |
| > 10 | > 30 | < 0.05 | < 0.2 | < 0.02 |

Additionally, sites were only retained if at least 1 of N samples with non-reference genotype had an allelic depth of > 0.3 to reduce false positives and potential mosaic calls.

**Canvas copy number calling**

Canvas CNV Caller (version 1.39.0.1598) was used to call copy number variation from WGS BAM files. Reference genomes and required supporting files were downloaded from <http://canvas-cnv-public.s3.amazonaws.com/> for GRCh38. Canvas calls were filtered for those with “PASS” in the VCF filter column and those intersecting with or containing the genomic loci for VHL, MET, FH, SDHB, SDHD, SDHC, BAP1, or CDKN2B.

Canvas command line script is found here: <https://github.com/maherlab/RCC_translocation/blob/master/canvas_cnv_calls.sh>

**Manta structural variant calling**

Manta Structural variant caller (version 1.3.1) was used to identify candidate chromosomal breakpoints matching cytogenetic banding and assess if structural variants had impacted on known RCC predisposition genes (VHL, MET, FH, SDHB, SDHD, SDHC, BAP1, or CDKN2B).

Manta command line script is found here: <https://github.com/maherlab/RCC_translocation/blob/master/manta_SV_calling.sh>

**Affymetrix OncoScan CNV FFPE array**

OncoScan CNV FFPE array was performed as described in the manfacturer’s protocol. SNP array data was analysed in the Chromosome Analysis Suite software (version 3.2; Applied Biosystems) using genome build GRCh37/Hg19.

Filters for artefactual CNV calls were performed by restricting calls to those consisting of a minimum of 50 consecutive probes and a minimum CNV length of 50 kilobases, with inclusion criteria for a list of whitelisted pathogenic alterations. Further analysis was performed by manual curation of the remaining CNV calls.

**GENE CODING REGIONS**

**Known RCC genes – BED file (GRCh38)**

| **Chromosome** | **Start** | **End** | **Gene** |
| --- | --- | --- | --- |
| chr3 | 52401013 | 52410350 | BAP1 |
| chr9 | 22002903 | 22009363 | CDKN2B |
| chr1 | 241497603 | 241519761 | FH |
| chr7 | 116672390 | 116798386 | MET |
| chr1 | 17018722 | 17054170 | SDHB |
| chr1 | 161314257 | 161375340 | SDHC |
| chr11 | 112086773 | 112120013 | SDHD |
| chr3 | 10141008 | 10152220 | VHL |

**SANGER SEQUENCING**

**Table 2 - PCR primers**

| **Translocation** | **Pair one (5’ to 3’)** | | **Pair two (5’ to 3’)** | |
| --- | --- | --- | --- | --- |
|  | **Left** | **Right** | **Left** | **Right** |
| t(2;17)(q21.1;q11.2) | TTCTGGCAGCGGGTCCA | CAAAAGGGCAGCAATGAACCA | TTCAATGATGTCATACTAGCAGCTT | GTGGACTTCAGGGAGATGCG |
| t(3;6)(p14.2;p12) | TCACCTGAAGTCTCTTCTTTCTT | CTCCAGGAAGTGATACATGGAA | GTCCTGTTTCCCTAGTCCTGC | AGGGAGGCAAGAAGGAAGTG |
| inv(3)(p21.1q12) | Failed to generate PCR products – 3 independent primer sets and nested primers | | | |
| t(3;14)(q13.3;q22) | CCCCAACAAACCCCACAACA | TGGACTCTGTATTCTGTTCCGT | GAGCTGAGATCATGCCATTGT | CTGAGTGGAGTCTGTATTTCCCA |
| t(10;17)(q11.22;p12) | GGCCACAATACTATGTCTCACC | ATACATGCGCACACAAGGTC | GGGACAGTGGAGAACGCAT | AAATTAGCTGGGCATGGTGG |

**IGV ALIGNMENTS**

**Figure 1 - t(2;17)(q21.1;q11.2)**


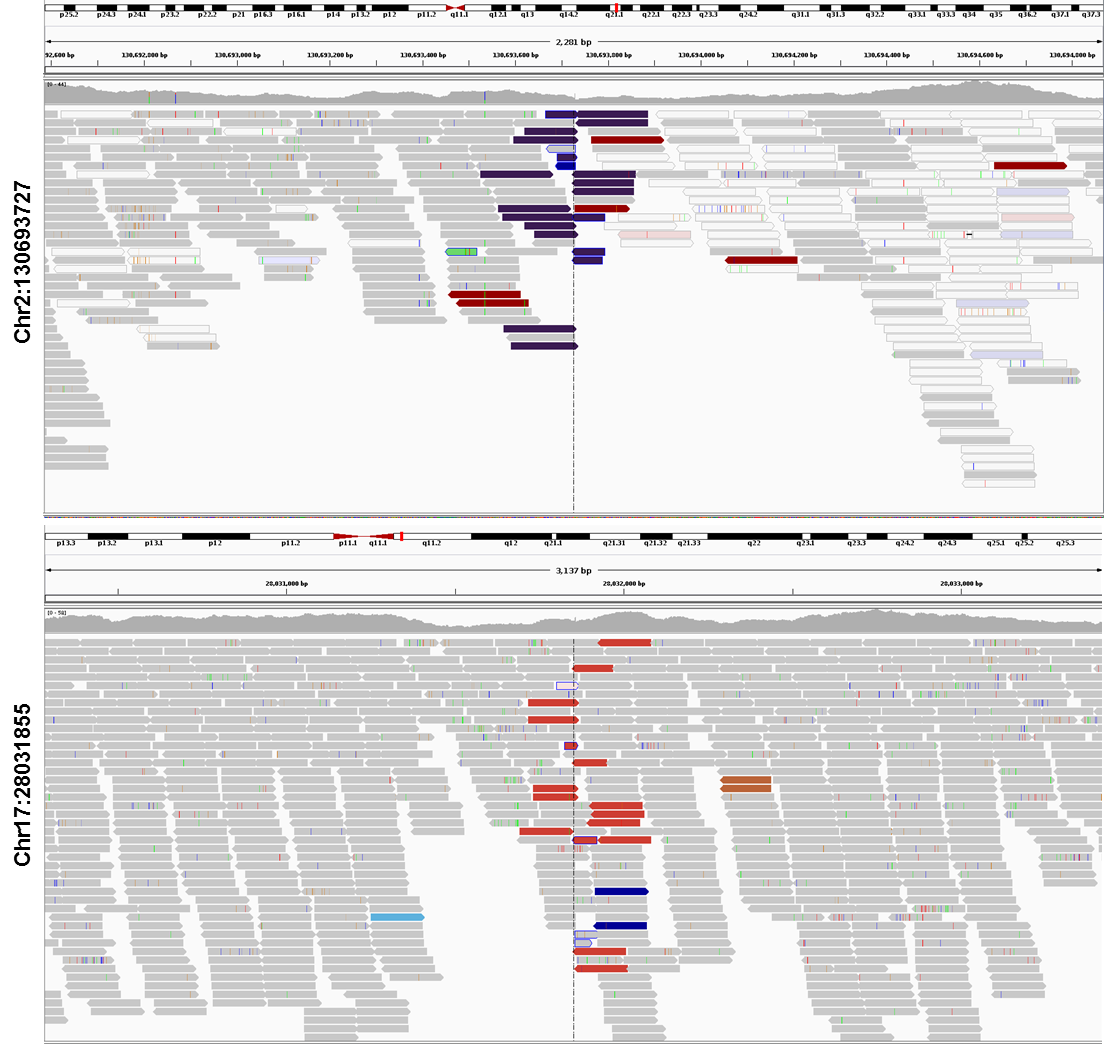


**Figure 2 - t(3;6)(p14.2;p12)**


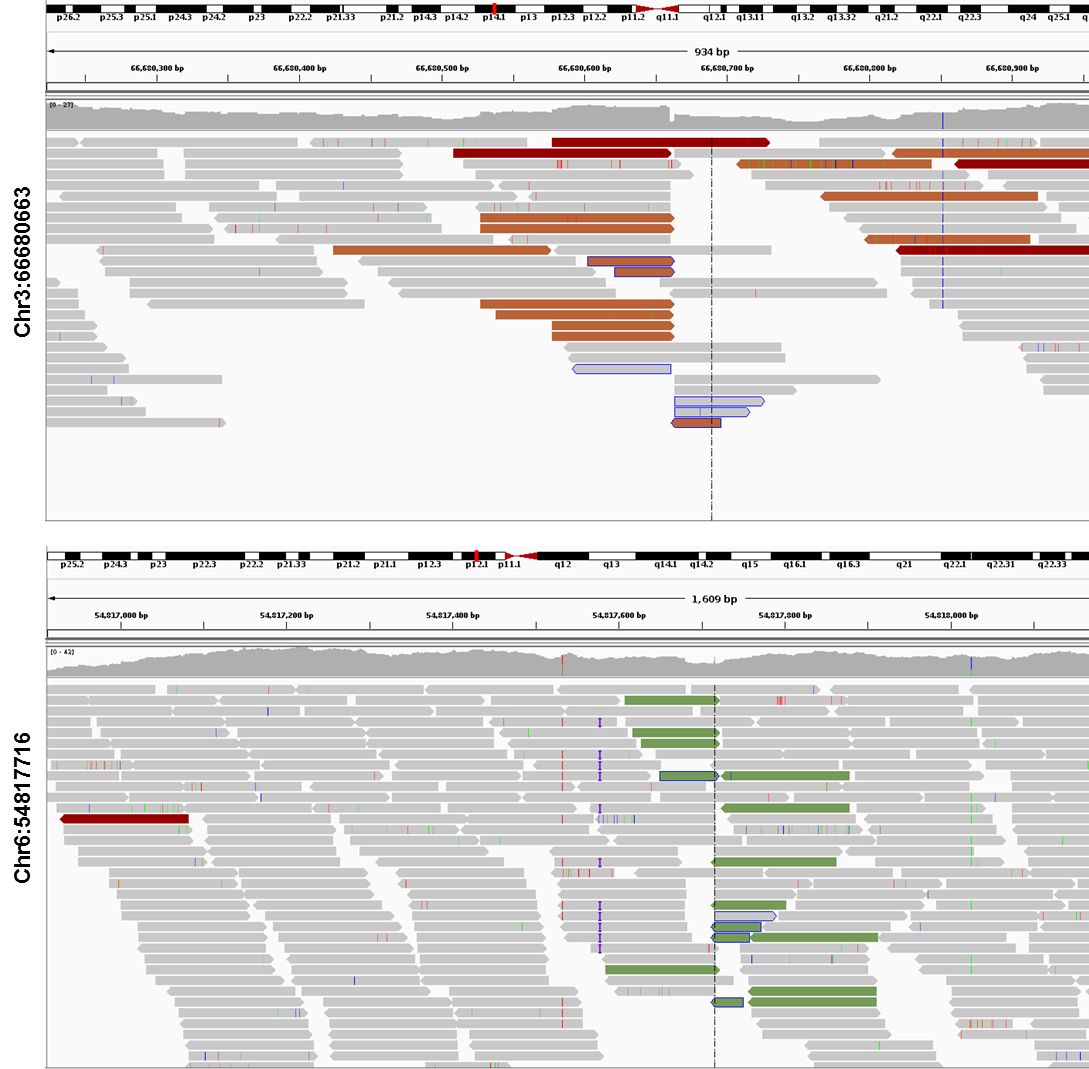


**Figure 3 - inv(3)(p21.1q12)**


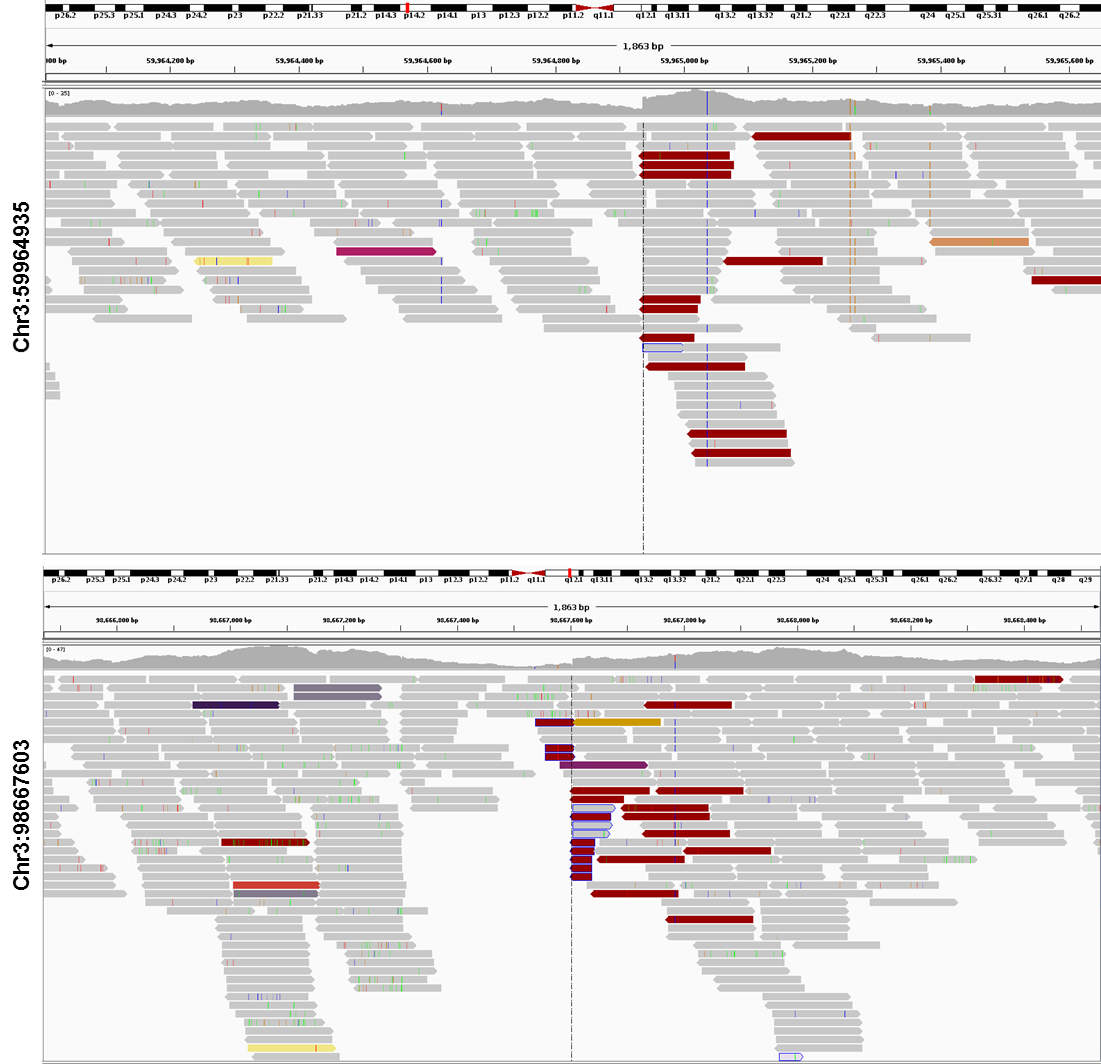


**Figure 4 - t(3;14)(q13.3;q22)**


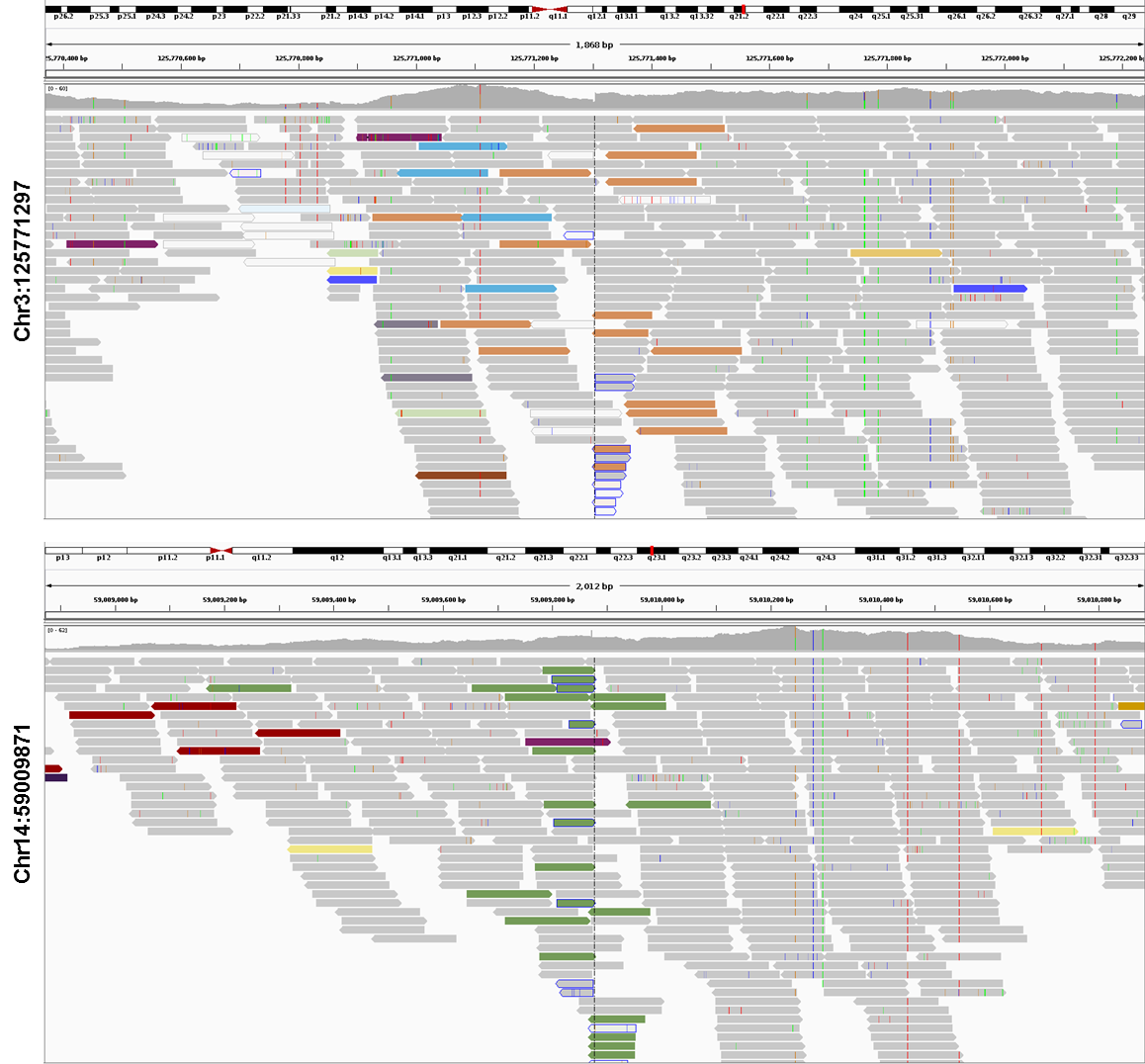


**
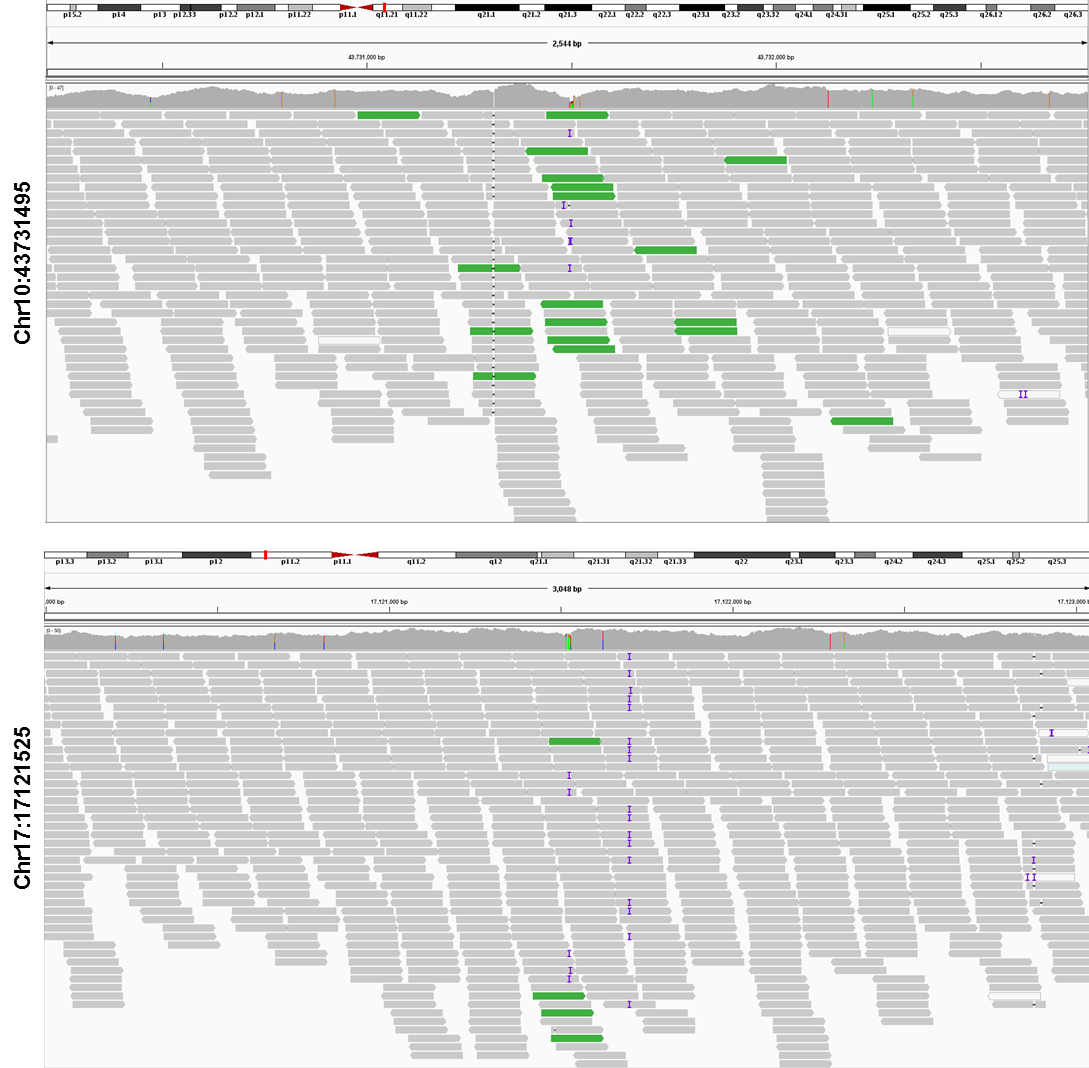
Figure 5 - t(10;17)(q11.22;p12)**

| **Case** | **CHR**  **A** | **START**  **A** | **END**  **A** | **CHR**  **B** | **START**  **B** | **END**  **B** | **QUAL** | **BND PAIR COUNT** | **PAIR COUNT** | **CIPOS** | **HOM**  **LEN** | **HOM**  **SEQ** | **JUNCT**  **QUAL** | **BND DEPTH** | **MATE BND DEPTH** | **FORMAT** | **SAMPLE** |
| --- | --- | --- | --- | --- | --- | --- | --- | --- | --- | --- | --- | --- | --- | --- | --- | --- | --- |
| t(2;17)  (q21.1;q11.2) | chr2 | 130693727 | 130693729 | chr17 | 28031855 | 28031856 | 491 | N/a | N/a | 0,1 | 1 | A | N/a | 19 | 37 | GT:FT:GQ  :PL:PR:SR | 0/1:PASS:233:  541,0,230:10,9:16,10 |
|  | chr17 | 28031855 | 28031857 | chr2 | 130693727 | 130693728 | 491 | N/a | N/a | 0,1 | 1 | T | N/a | 37 | 19 | GT:FT:GQ  :PL:PR:SR | 0/1:PASS:233:  541,0,230:10,9:16,10 |
| t(3;6)  (p14.2;p12) | chr3 | 66680663 | 66680664 | chr6 | 54817716 | 54817717 | 837 | N/a | N/a | N/a | N/a | N/a | 227 | 21 | 30 | GT:FT:GQ  :PL:PR:SR | 0/1:PASS:383:  887,0,380:5,4:12,7 |
|  | chr6 | 54817716 | 54817717 | chr3 | 66680663 | 66680664 | 837 | N/a | N/a | N/a | N/a | N/a | 227 | 30 | 21 | GT:FT:GQ  :PL:PR:SR | 0/1:PASS:383:  887,0,380:5,4:12,7 |
| t(3;14)  (q13.3;q22) | chr3 | 125771297 | 125771301 | chr14 | 59009871 | 59009872 | 999 | N/a | N/a | 0,3 | 3 | ATG | 333 | 40 | 50 | GT:FT:GQ  :PL:PR:SR | 0/1:PASS:941:  999,0,938:15,7:29,9 |
|  | chr14 | 59009871 | 59009875 | chr3 | 125771297 | 125771298 | 999 | N/a | N/a | 0,3 | 3 | TGT | 333 | 50 | 40 | GT:FT:GQ  :PL:PR:SR | 0/1:PASS:941:  999,0,938:15,7:29,9 |
| t(10;17)  (q11.22;p12) | chr10 | 43236058 | 43236059 | chr17 | 17218216 | 17218217 | 1602 | 16 | 16 | N/a | N/a | N/a | 914 | 39 | 59 | GT:GQ:  PR:SR | 0/1:1602:  35,15:18,18 |
|  | chr17 | 17218216 | 17218217 | chr10 | 43236058 | 43236059 | 1602 | 16 | 16 | N/a | N/a | N/a | 914 | 59 | 39 | GT:GQ:  PR:SR | 0/1:1602:  35,15:18,18 |
|  | chr10 | 43236047 | 43236050 | chr17 | 17218213 | 17218214 | 1602 | 22 | 22 | 0,2 | 2 | TG | 637 | 39 | 58 | GT:GQ:  PR:SR | 0/1:1602:  36,22:17,10 |
|  | chr17 | 17218211 | 17218214 | chr10 | 43236049 | 43236050 | 1602 | 22 | 22 | 0,2 | 2 | CA | 637 | 58 | 39 | GT:GQ:  PR:SR | 0/1:1602:  36,22:17,10 |
| **Case** | **CHR**  **A** | **START**  **A** | **END**  **A** | **CHR**  **B** | **START**  **B** | **END**  **B** | **QUAL** | **END** | | | **SVLEN** | | | **FORMAT** | | | **SAMPLE** |
| inv(3)  (p21.1q12) | chr3 | 59964935 | 59964936 | chr3 | 98667603 | 98667604 | 602 | 98667604 | | | 38702668 | | | GT:FT:GQ  :PL:PR:SR | | | 0/1:PASS:93:  652,0,90:2,11:10,11 |

**Table 3 - Manta calls supporting translocation and inversion breakpoints**

**Figure 6 – Sanger sequencing traces supporting chromosomal translocation breakpoints**


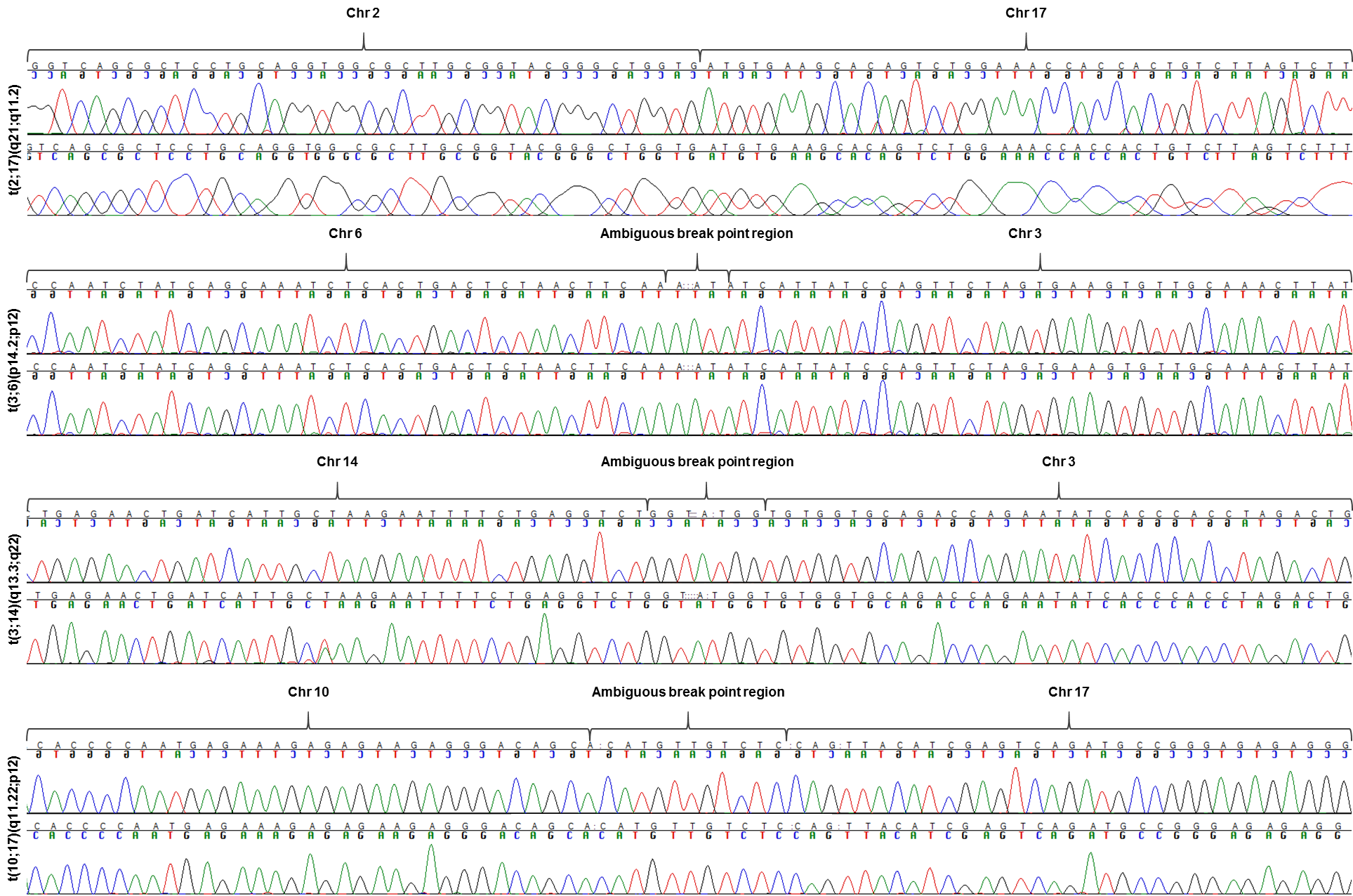


**Ambiguous breakpoint region**

**Ambiguous breakpoint region**

**Ambiguous breakpoint region**

**Figure 7 – TAD analysis t(3;14)(q13.3;q22) - chr14:59009871**

**
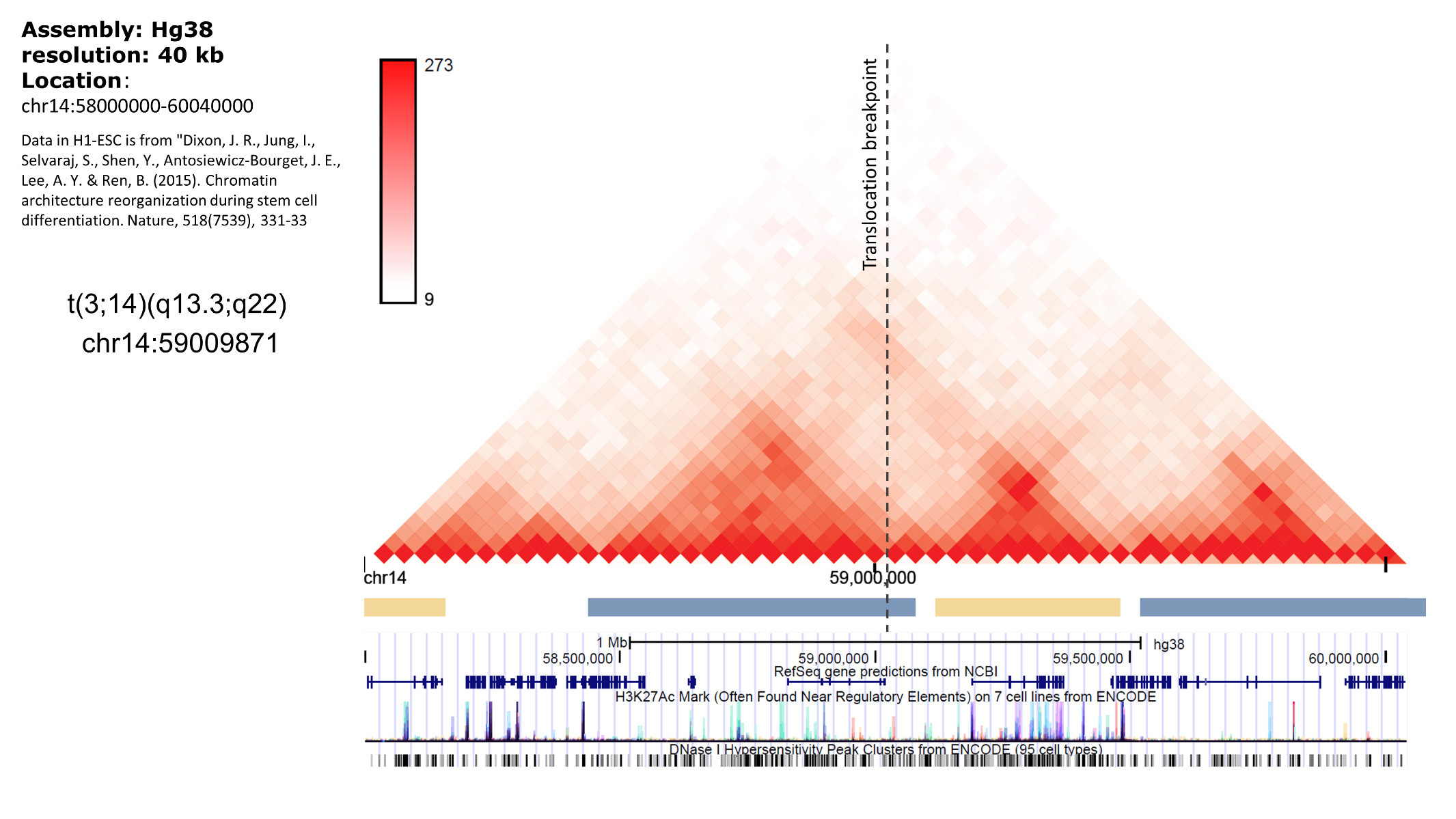
**

**Figure 8 – TAD analysis t(3;14)(q13.3;q22) - chr3:125771297**

**
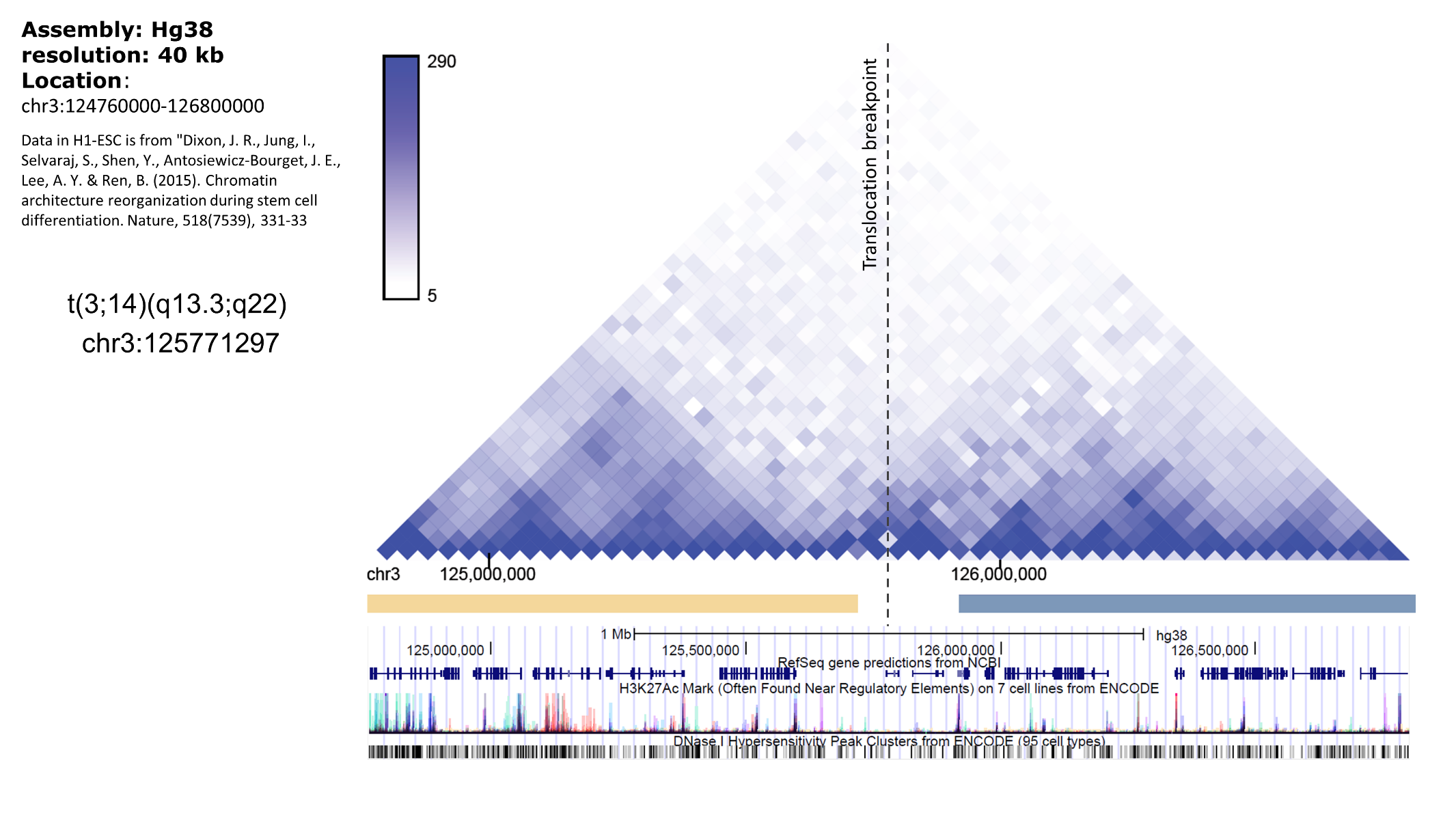
**

**Figure 9 – TAD analysis t(3;6)(p14.2;p12) - chr3:66680663**

**
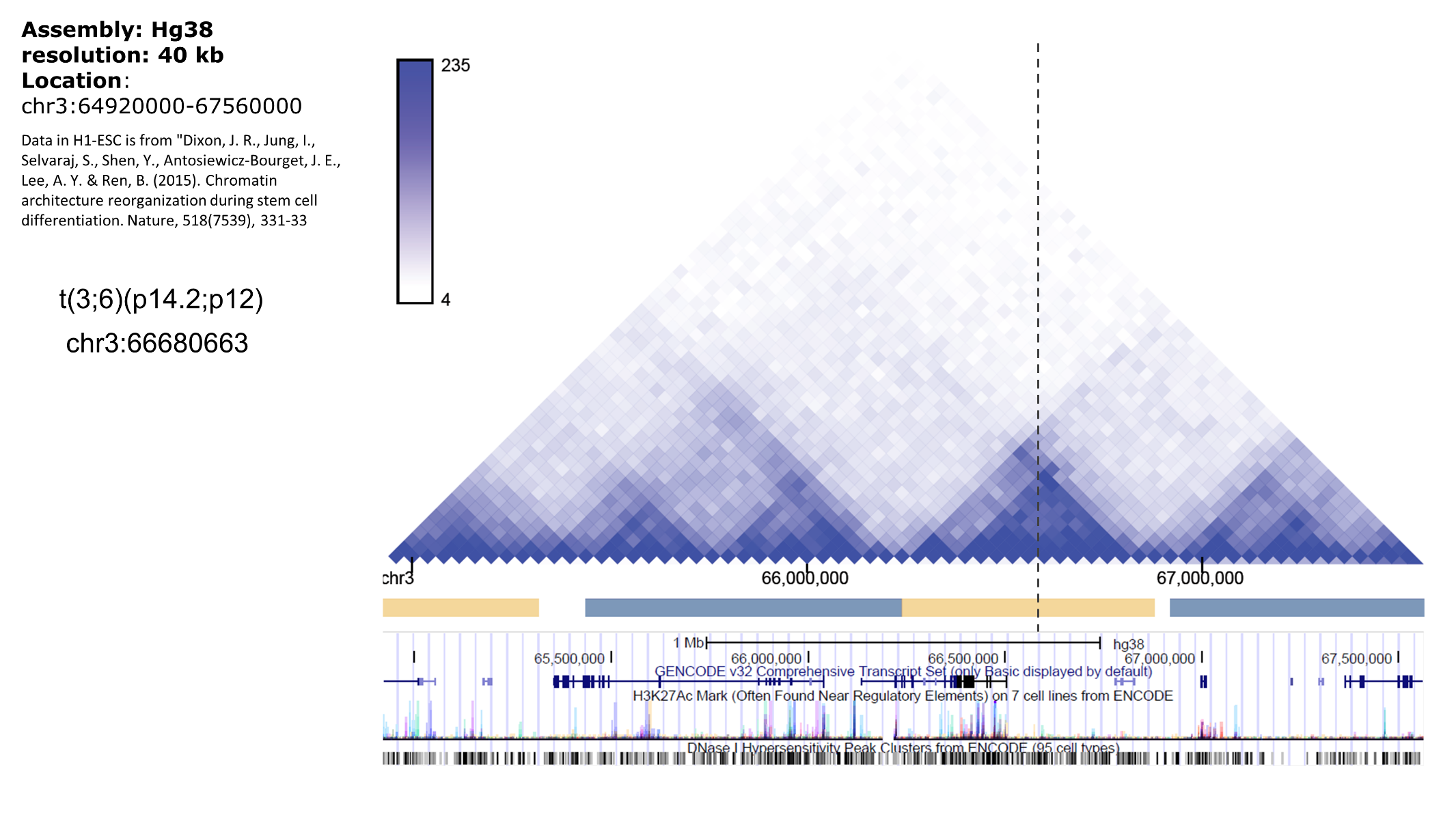
**

**Figure 10 – TAD analysis t(3;6)(p14.2;p12) - chr6:54817716**

**
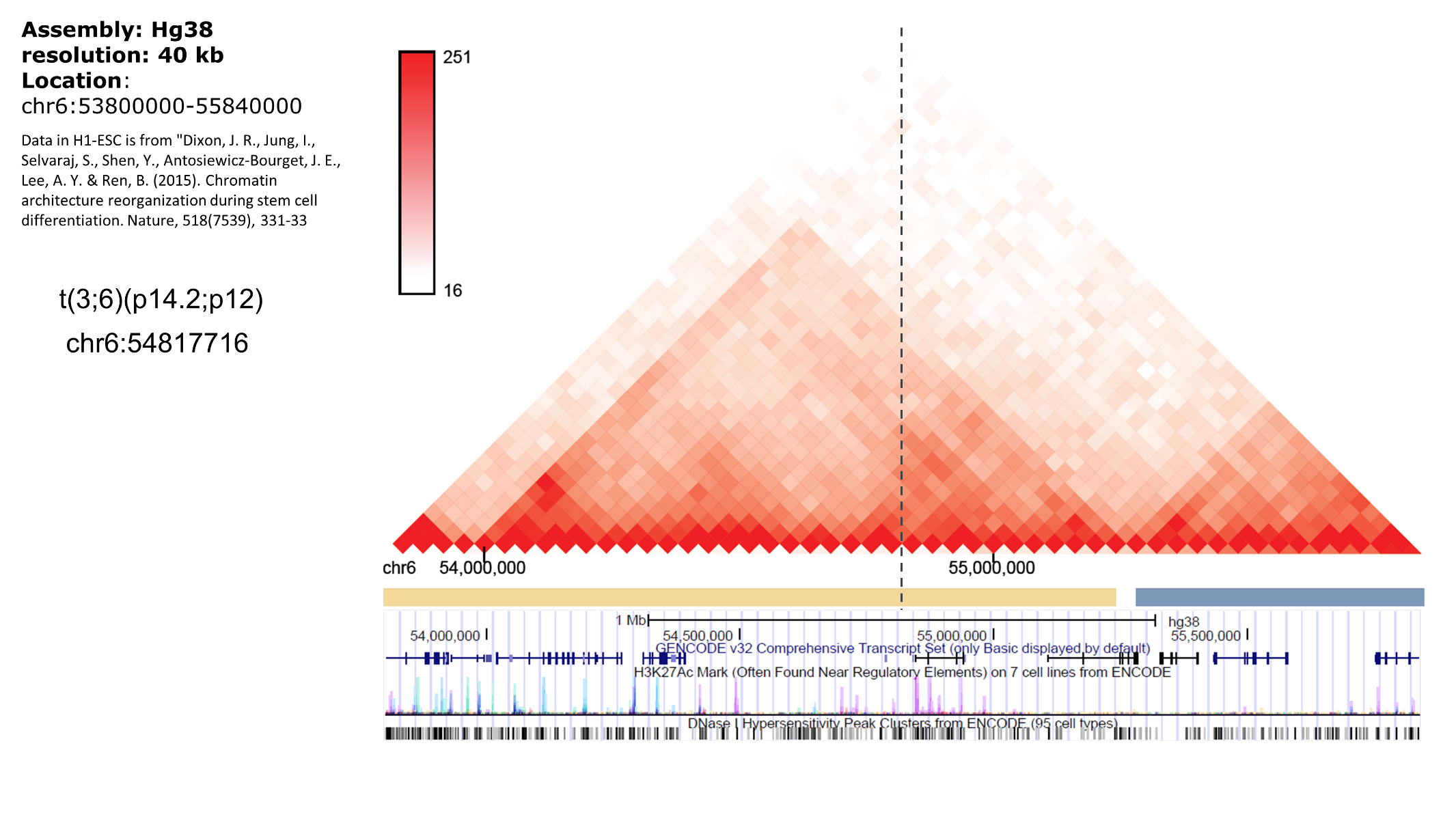
**

**Figure 11 – TAD analysis inv(3)(p21.1q12) - chr3:59964935**

**
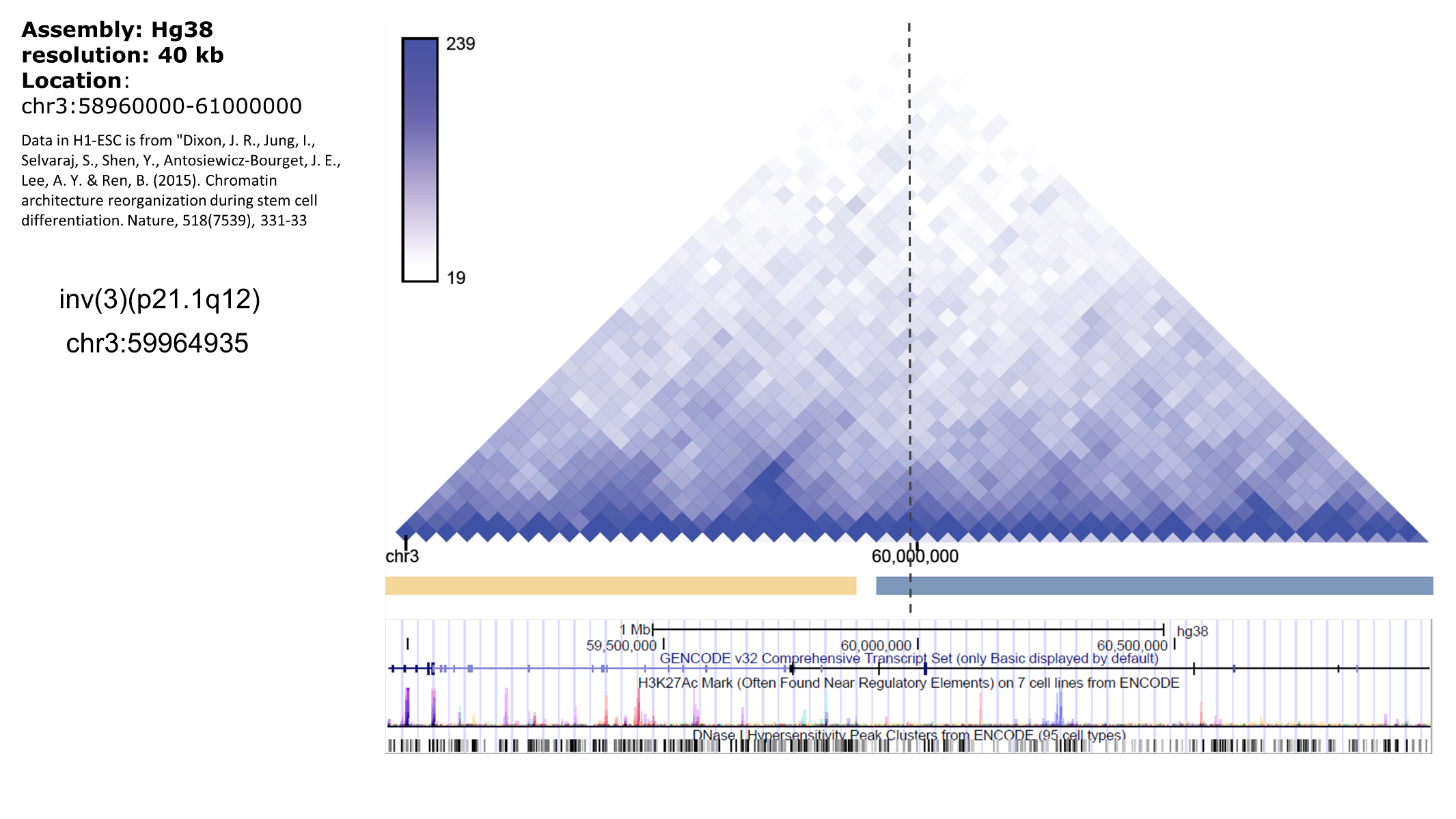
**

**Figure 12 – TAD analysis inv(3)(p21.1q12) - chr3:98667603
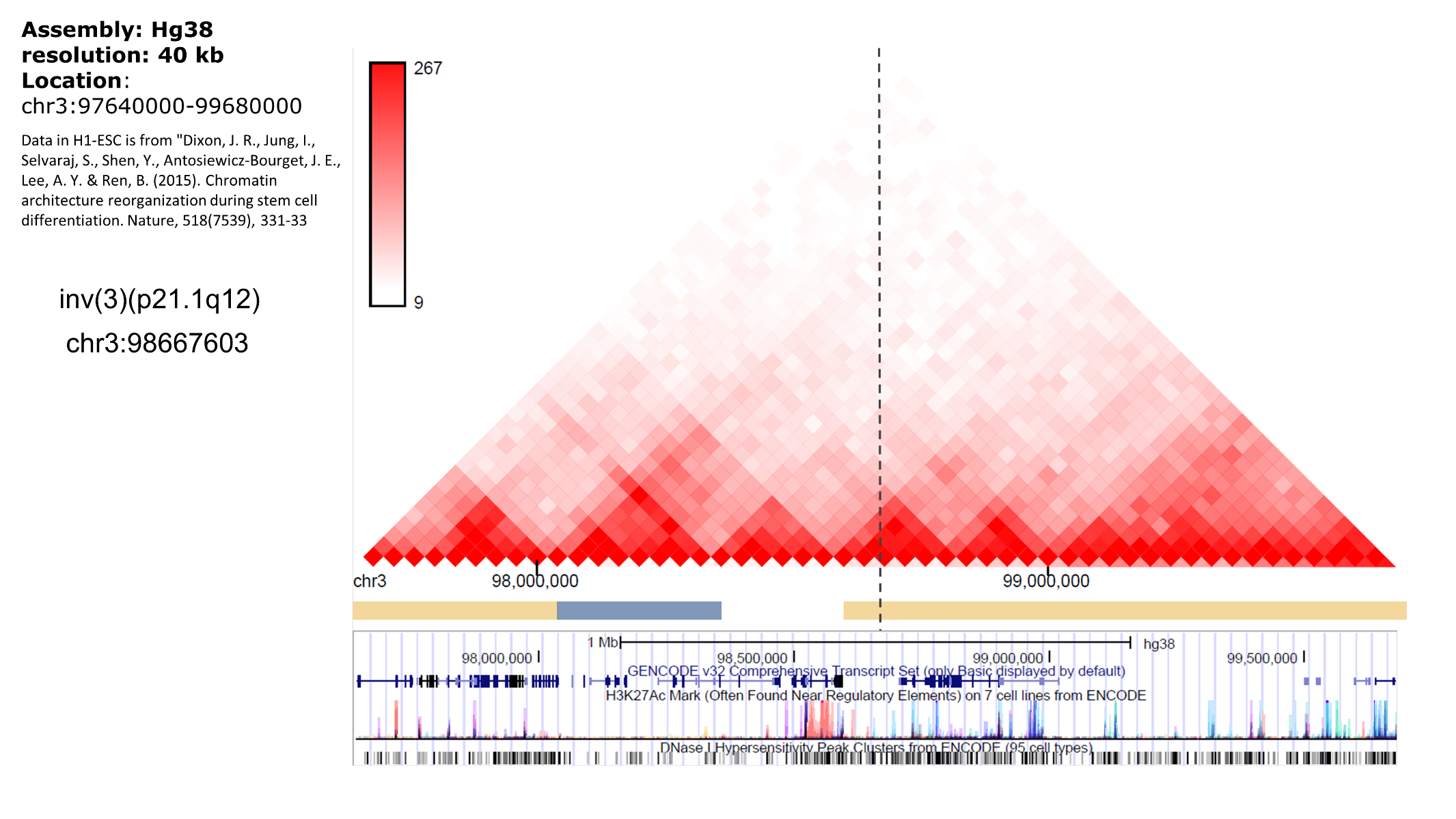
**

**Figure 13 – TAD analysis t(2;17)(q21.1;q11.2) - chr2:130693728**

**
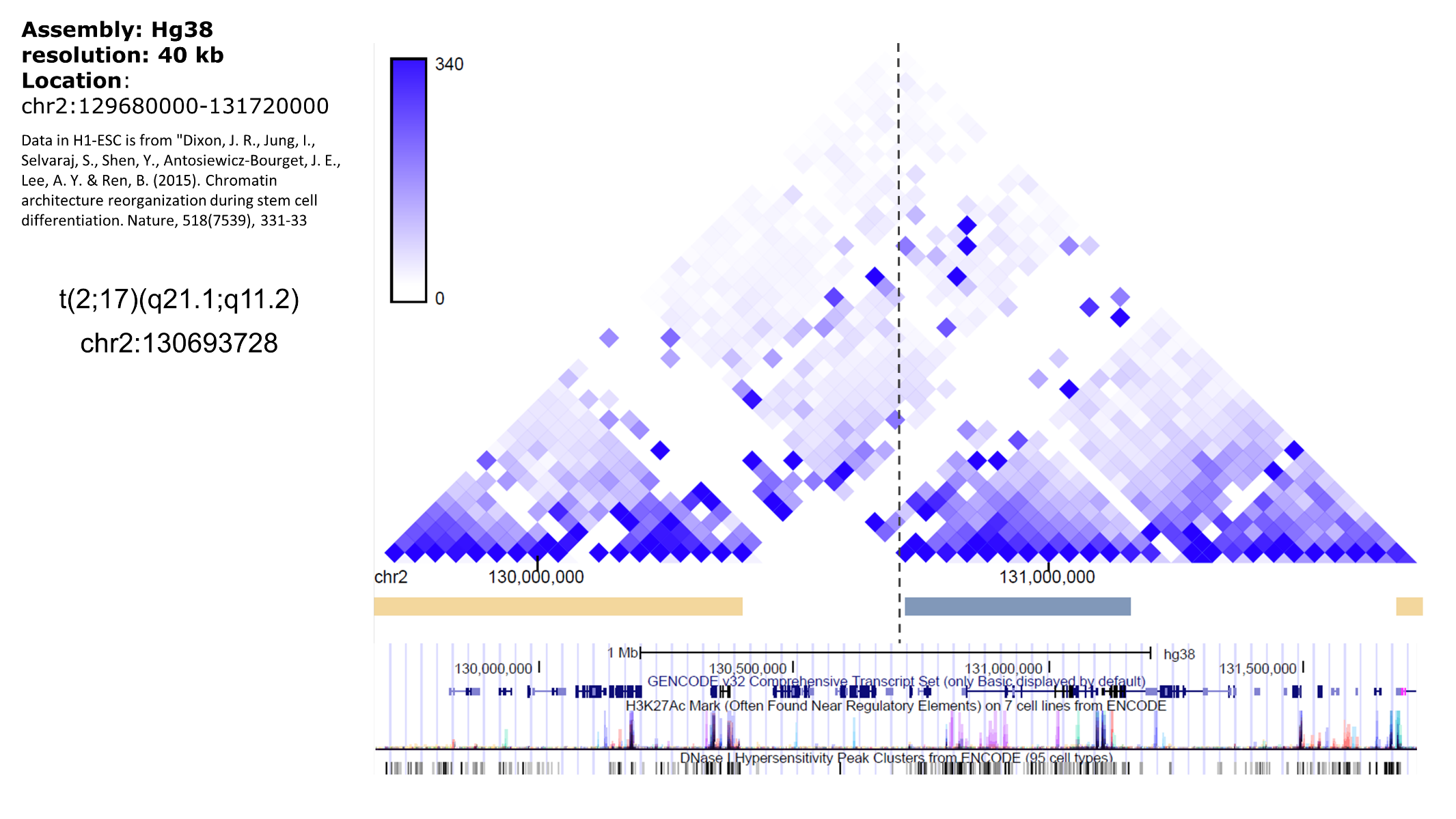
**

**Figure 14 – TAD analysis t(2;17)(q21.1;q11.2) - chr17:28030855**

**
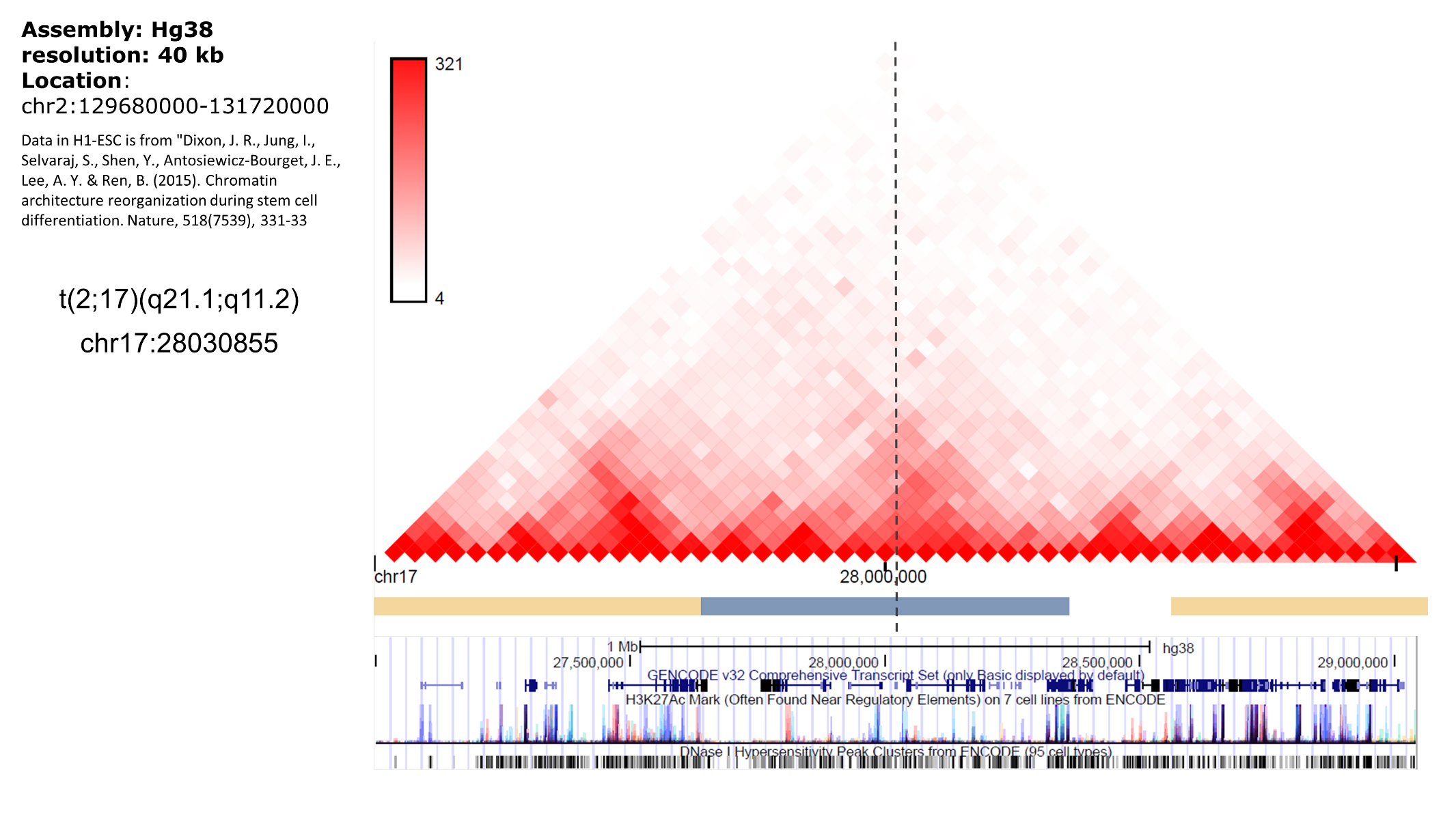
**

**Figure 15 – TAD analysis t(10;17)(q11.22;p12) - chr17:17218211
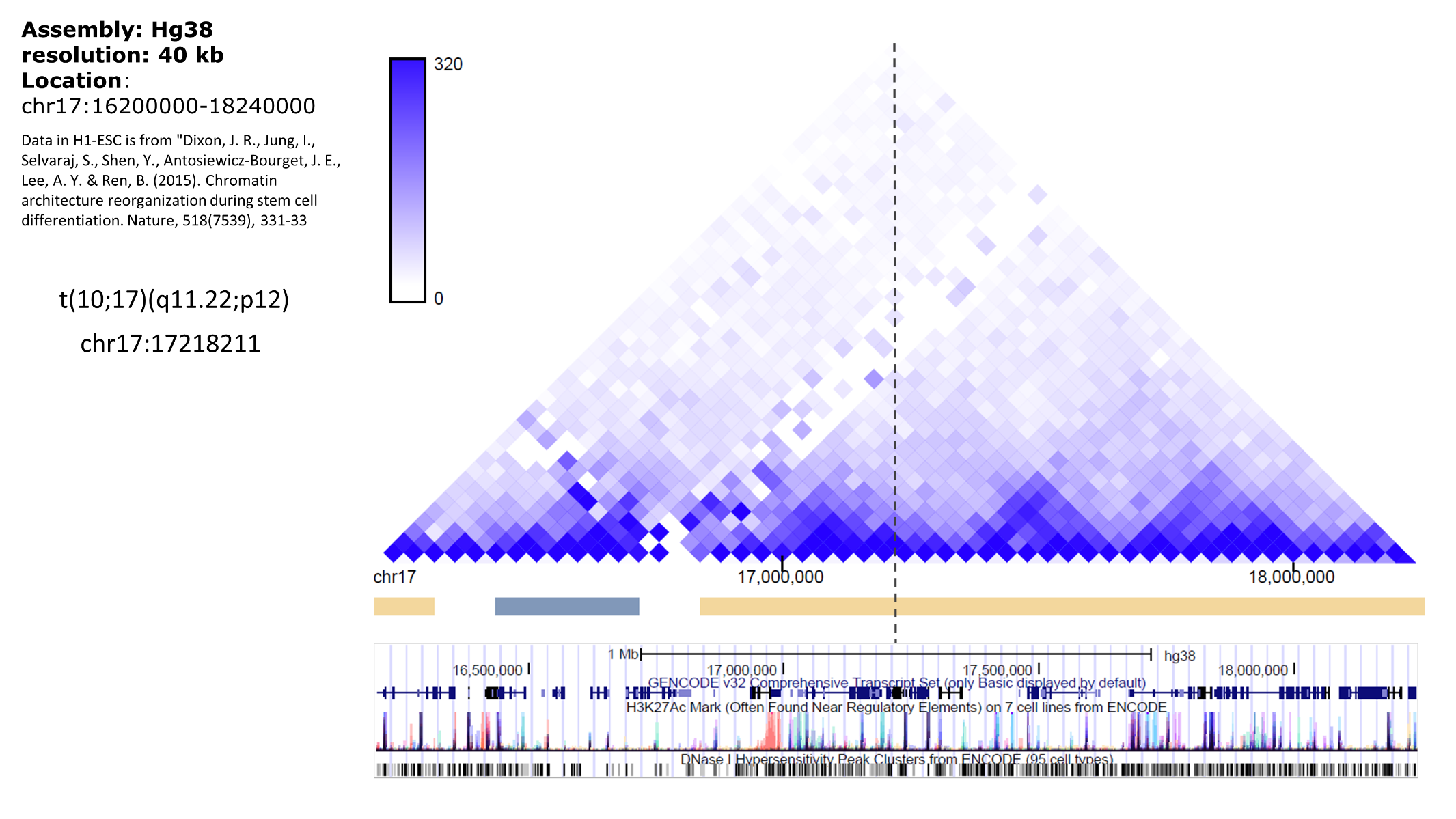
**

**Figure 16 – TAD analysis t(10;17)(q11.22;p12) - chr10:43236047**

**
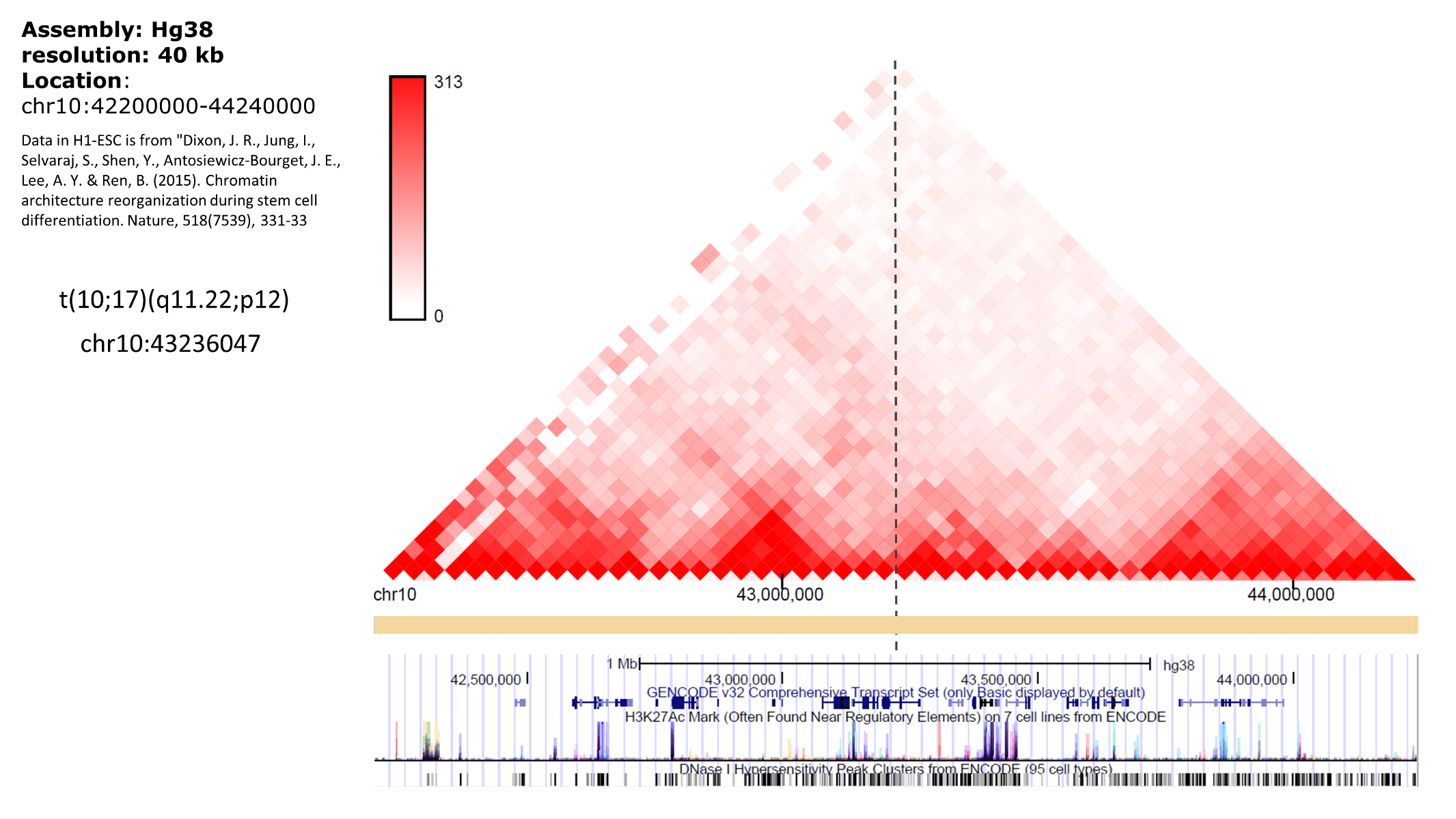
**

**Figure 17 – Affymetrix OncoScan CNV FFPE array – der(3)(0 - 198,022,430) Log2 Ratio, copy number state and BAF**


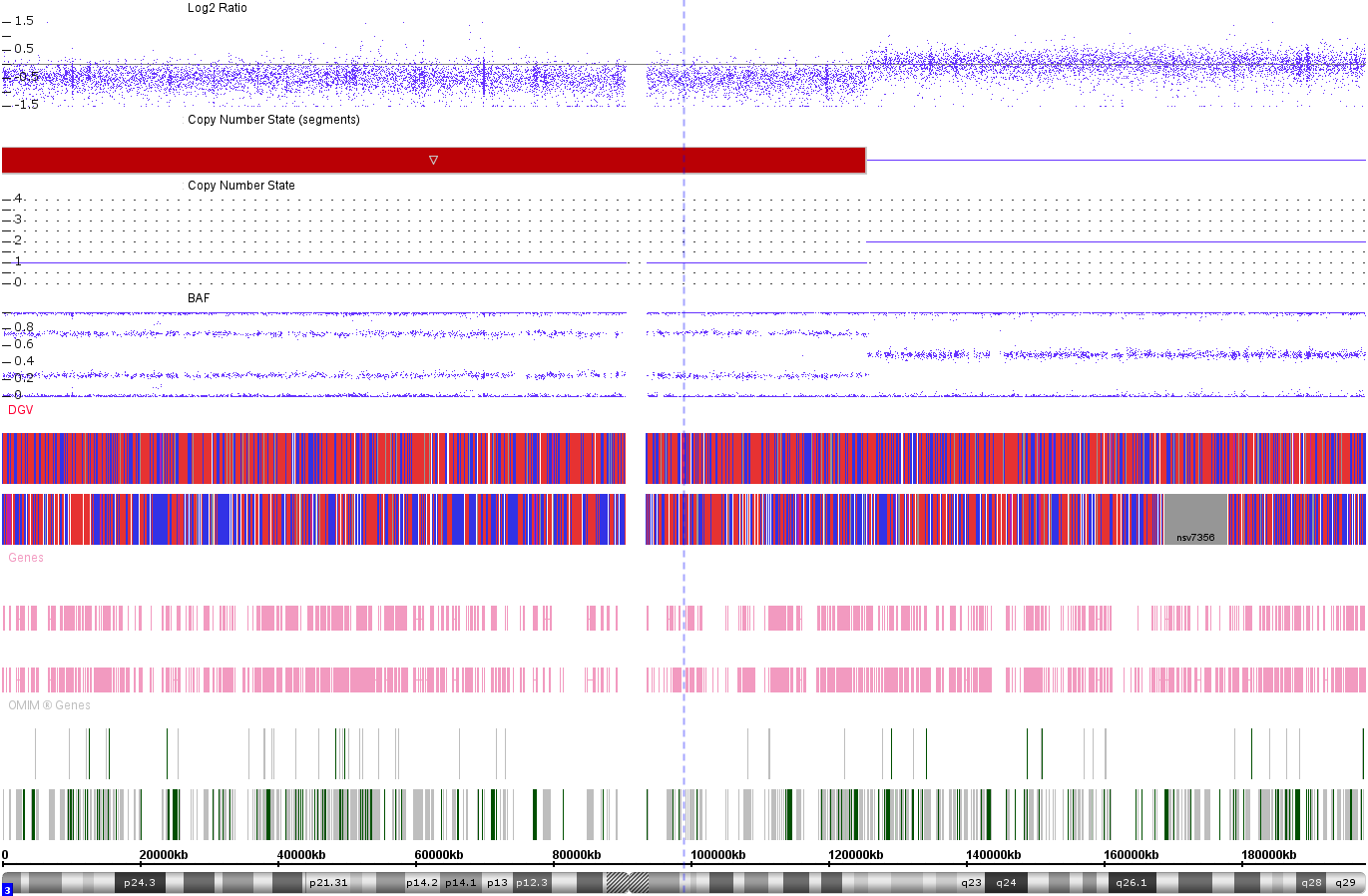


**Figure 17 – Affymetrix OncoScan CNV FFPE array – der(14)(0 – 107,349,540) Log2 Ratio, copy number state and BAF**
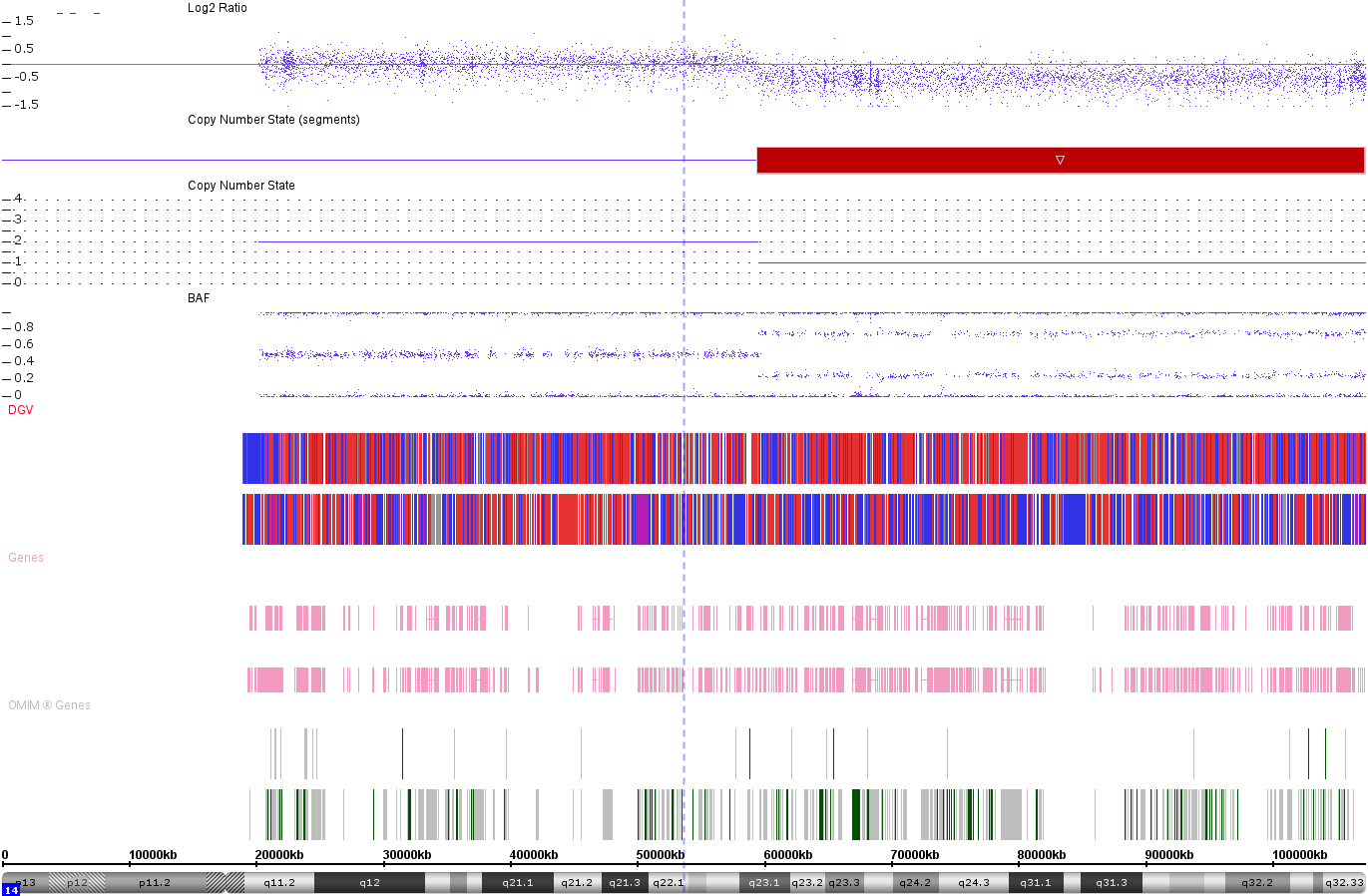

Supplement: Supplementary file 1 — Appendix S1: Supporting Information [file GCC-59-333-s001.docx]
